# Grid-Based Path Planning of Agricultural Robots Driven by Multi-Strategy Collaborative Evolution Honey Badger Algorithm

**DOI:** 10.3390/biomimetics10080535

**Published:** 2025-08-14

**Authors:** Yunyu Hu, Peng Shao

**Affiliations:** School of Computer and Information Engineering, Jiangxi Agricultural University, Nanchang 330000, China; yunyuh@stu.jxau.edu.cn

**Keywords:** honey badger optimisation algorithm, mobile robot path planning, grid method, centre of gravity inverse learning, differential evolution strategy

## Abstract

To address the limitations of mobile robots in path planning within farmland-specific environments, this paper proposes a biomimetic model: Multi-strategy Collaborative Evolution Honey Badger Algorithm (MCEHBA), MCEHBA achieves improvements through the following strategies: firstly, integrating a sinusoidal function-based nonlinear convergence factor to dynamically balance global exploration and local exploitation; secondly, combining the differential evolution strategy to enhance population diversity, and utilizing gravity-centred opposition-based learning to improve solution space search efficiency; finally, constructing good point set initialization and decentralized boundary constraint handling strategy to further increase convergence accuracy and speed. This paper validates the effectiveness of the optimization strategy and the performance of MCEHBA through the CEC2017 benchmark function set, and assesses the statistical significance of the results using the Friedman test and Nemenyi test. The findings demonstrate that MCEHBA exhibits excellent optimization capabilities. Additionally, this study applied MCEHBA to solve three engineering application problems and compared its results with six other algorithms, showing that MCEHBA achieved the minimum objective function values in all three cases. Finally, simulation experiments were conducted in three farmland scenarios of varying scales, with comparative tests against three state-of-the-art algorithms. The results indicate that MCEHBA generates paths with minimized total costs, demonstrating superior global convergence and engineering applicability.

## 1. Introduction

With the rapid growth of the global population, the provision of adequate food for a large population has put some pressure on the relevant sectors [1]. Global food production still needs to be increased to achieve the vision of “zero hunger” in the United Nations Sustainable Development Goals. Modern agricultural operations encompass links, including crop cultivation, plant protection management, harvesting and transport, requiring workers to traverse farmlands or greenhouses. Furthermore, these operations are characterized by high repetitiveness, long operating hours and high energy consumption on workers, and this high-intensity labor inherently limits operational efficiency due to physiological constraints. Consequently, the daily output often fails to meet market demands. Therefore, improving the efficiency of agricultural operations has become key to addressing these challenges.

Mobile robots, currently a major research subject for scientists [2], are steadily maturing. Present research topics mainly include navigation and localisation, motion control and path planning [3]. The path planning algorithm was first applied in the autonomous mobile robot developed by Stanford Research Institute (SRI) and has been rapidly developed in recent years, becoming a key research area in the field of robotics and artificial intelligence [4]. Some experiments have pointed out that mechanical harvesting achieves three times the efficiency of manual harvesting [5]. Combining innovative technologies with traditional practices by leveraging mobile robots to enhance agricultural capabilities can effectively improve operational efficiency, and this approach provides an innovative solution to address the contradiction between the global population growth and the scarcity of arable land resources and the limited manpower. However, the high number of obstacles such as barns and fields pose a serious challenge to the autonomous navigation capability of the mobile robot, inaccurate path planning will directly affect the operational efficiency and may cause crop damage and economic losses. Therefore, how to find a reasonable and effective path for the robot is an issue worth exploring. In addition, an adapted environment model can effectively represent the obstacles to achieve the maximum restoration of the real scene, reduce unnecessary planning. Environment modelling refers to the abstraction of real-world scenarios into obstacle fields using different environment modelling methods, and an obstacle field is a virtual field formed by all obstacles and their surrounding area of influence in the workspace of an object such as a robot; it is mainly presented in the form of a diagram, which is used to describe the obstacles that exist in the space.

In order to address both of these issues, we first identified the grid method as the method for modelling the environment for the following reasons: The global path planning studied in this paper has the characteristics that the obstacle field is known and fixed, so what is obtained from the environment modelling will be a two-dimensional map that will not change. The main modelling methods that are currently considered effective and widely used are the visual graph method, the Voronoi diagram method, the topology method and the grid method. Given the intrinsic compatibility between the grid method and agricultural environments—where typical scenarios such as farmland, orchards, and greenhouse facilities exhibit regular row-column layouts that facilitate grid discretization, this study adopts the grid-based method for environmental modelling.

The grid method discretizes the environment into uniformly spaced grid cells, and maps the real-world environment onto a grid map. Each grid has two states of passable and impassable, which can be expressed by 0 or 1, and the starting point and the end point each occupy a grid. The grid method is intuitive and highly adaptable. By dividing the environment into grids, path planning resembles moving pieces on a chessboard. This approach can be effectively implemented regardless of environmental regularity, provided the traversability of grid cells is accurately assessed. It offers high computational efficiency and optimisation potential. However, grid size affects planning precision. Excessively large grids reduce path accuracy, while excessively small grids increase computational load and processing time; this necessitates careful calibration of the grid division—the most significant drawback identified in our analysis.

In recent years, bio-inspired algorithms have been widely applied to solve complex engineering problems due to their powerful optimisation capabilities. These algorithms are often inspired by the behaviour of organisms in nature. They conceptualise optimisation problems by analogy with natural processes, such as biological evolution, group foraging, immune responses, or neural activity. Key mechanisms from these processes—including selection, crossover, mutation, pheromone deposition, and collective collaboration—are then simulated to find solutions. Prominent examples include Genetic Algorithms (GA) [6], which simulate biological evolution; Particle Swarm Optimisation (PSO) [7], inspired by bird flocking; and Ant Colony Optimisation (ACO) [8], based on ant foraging behaviour. These algorithms possess strong global search capabilities, adaptability to complex non-linear problems, and robustness. They are particularly adept at finding satisfactory solutions within high-dimensional, constrained environments. These characteristics align closely with the core challenges of mobile robot path planning. The robot must generate a safe, smooth, and energy-optimal movement trajectory while avoiding static and dynamic obstacles. It must also satisfy its own kinematic constraints, such as minimum turning radius and speed limits, whilst dealing with sensor noise and environmental modelling uncertainties. Through their inherent parallel exploration mechanisms and intelligent decision-making strategies, bio-inspired algorithms provide a naturally well-suited framework for addressing this series of complex challenges.

For the implementation difficulties of path planning in complex environments, the existing traditional intelligent optimisation algorithms generally face bottlenecks such as slow convergence speed and local optimal traps. To this end, researchers have proposed a variety of new intelligent optimisation algorithms and their improved versions. Qiu G et al. proposes a Hybrid Clustering-Enhanced Brain Storm Optimization (HC-BSO) algorithm designed to improve both path quality and computational efficiency significantly [9]; Yun O et al. proposed a hybrid grey wolf optimisation algorithm, pGWO-CSA, which was employed for mobile robot path planning and achieved favourable results [10]; Yang Z et al. enhanced the traditional Particle Swarm Optimisation algorithm using an improved sine chaos map, quantum mechanics concepts, and Lévy flight strategies. The resulting improved algorithm, applied to path planning, succeeds in minimising algorithmic complexity while maximising search precision [11]. These improved algorithms outperform the traditional methods in terms of convergence speed and solution accuracy. Accordingly, in this paper, a scheme based on honey badger optimisation algorithm is designed to solve the problem of mobile robot path planning.

Honey Badger Algorithm is a new swarm intelligence algorithm proposed by Hashim et al. in 2022 to solve various optimisation problems [12], similar to the idea of other intelligent optimisation algorithms such as Whale Optimisation Algorithm (WOA) [13] and Ant Colony Algorithm (ACO) [14], which is inspired by animals in nature, and the core idea is to simulate a honey badger in search of food (such as honey or insects) when it exhibits the behaviours of exploration (global search) and exploitation (local search), and solves complex optimization problems by dynamically balancing these two strategies, which has the characteristics of simple structure, fewer parameters, fast convergence speed, and strong local search ability, and has been welcomed by a wide range of researchers. Ajay et al. used HBA to optimise the configuration and sensitivity analysis of a multi-objective optimisation problem and proved that HBA outperforms other good algorithms [15]; Jia et al. used a lens imaging reverse learning strategy to enhance the exploration capability of the algorithm and applied it to the UAV 3D path planning problem [16]; Geetha et al. investigated the problem of energy-efficient hybrid flow shop (EEHFS) scheduling using a hybrid Honey Badger Algorithm (HHBA) [17].

Simultaneously, HBA exhibits certain limitations: its global exploration capability is relatively weak, and it tends to stagnate in local optima when addressing highly complex multimodal problems. To mitigate these constraints and enhance path planning efficacy for mobile robots, this paper introduces multiple enhancement strategies based on the conventional Honey Badger Algorithm. We propose a Multi-strategy Collaborative Evolution Honey Badger Algorithm (MCEHBA), incorporating five key mechanisms: good point set initialisation, decentralized boundary constraint-handling strategy, sinusoidal function-based nonlinear convergence factor, differential evolution strategy, and gravity-centredopposition-based learning. This specific combination of strategies has not been previously documented in the literature. Moreover, the integration is purposefully structured rather than a simple stacking of techniques, it constitutes a systematic and targeted design to address critical challenges in complex optimization problems. This approach identifies superior solutions with heightened convergence precision, ultimately generating safer and more cost-efficient paths for mobile robots.

The paper is structured as follows: Section 2 reviews related work, describing fundamental principles of the Honey Badger Algorithm (HBA) and its enhancement strategies; Section 3 details the algorithm design and optimisation process, analyses the mechanistic role of improvements, and provides pseudocode. Section 4 experimentally validates the enhanced algorithm through CEC2017 benchmark function verification and comparative evaluations against state-of-the-art algorithms, with statistical significance ensured via Friedman and Nemenyi tests. Convergence behaviour of MCEHBA is also examined, alongside practical validation using three multi-scenario engineering case studies. Section 5 applies MCEHBA to path planning, defining experimental procedures and objective functions while designing three distinct planning scenarios with quantitative results analysis. Section 6 discusses key experimental conclusions, and finally Section 7 summarises findings while outlining future applications and improvement directions for MCEHBA in optimisation problems.

## 2. Related Work

### 2.1. Honey Badger Optimisation Algorithm

Next, we will detail the core implementation process and key mathematical models of Honey Badger Algorithm (HBA). This algorithm introduces seven random parameters ($r_{1}$ to $r_{7}$), all defined within the open interval (0,1). The random number r6 solely determines the value of the directional variable F, while the remaining six random variables enhance the algorithm’s exploration capability through controlled stochastic perturbations.

#### 2.1.1. Population Initialisation

In the feasible solution space, the position of each individual in the population is initialised and represented as a two-dimensional matrix as follows. The matrix’s row index corresponds to the individual identifier within the population, while the column index denotes the dimension index of the solution vector, where N specifies the population size (number of individuals) and D indicates the dimensionality of the optimization problem.

$$position=[\begin{array}{ccccc} x_{11} & x_{12} & x_{13} & \cdots & x_{1D}\\ x_{21} & x_{22} & x_{23} & \cdots & x_{2D}\\ & \cdots & \cdots \cdots & \cdots & \\ x_{N1} & x_{N2} & x_{N3} & \cdots & x_{ND} \end{array}]$$

The position initialization for each honey badger is formulated as follows:

(1) $$x_{i}=lb_{i}+r_{1}\times (ub_{i}+lb_{i}).$$

Here, ${\;x}_{i\;}$ denotes the position of the ith honey badger, ${lb}_{i}$ represents the lower boundary of the activity range for the ith honey badger, ${\;ub}_{i}$ signifies the upper boundary of the activity range for the ith honey badger.

#### 2.1.2. Odour Intensity Setting

The odour intensity influences the activity speed of honey badger individuals in excavation mode. When the target’s odour is strong, honey badgers can detect the target more rapidly. The odour intensity is related to both the distance between the honey badger and the target and the aggregation degree among honey badgers. The update equation for odour intensity is given based on the inverse square law.

(2) $$I_{i}=r_{2}\times \frac{S}{4\pi d_{i}^{2}},$$

(3) $$\begin{array}{cc} S & ={\left(x_{i}-x_{i+1}\right)}^{2} \end{array},$$

(4) $$d_{i}=x_{prey}-x_{i}.$$

Here, $X_{prey}$ represents the target position (assigned to the individual with the optimal fitness value within the population).

#### 2.1.3. Density Factor Setting

To ensure smooth transition from exploration to exploitation, a density factor α is introduced, which decays exponentially over iterations:

(5) $$\alpha =C\times \exp\left(\frac{-t}{t_{max}}\right),$$

where C is an empirically determined constant (optimized as C=2 through parametric studies in the original work), t denotes the current iteration index, and $t_{max}$ specifies the maximum iteration threshold. This decay mechanism progressively shifts algorithmic focus from global search to local refinement.

#### 2.1.4. Digging Mode

In the digging mode, the honey badger is influenced by the intensity of the scent to hover either near or far from the target, and its action trajectory is similar to a cardioid-shaped, with the prey located in the centre of the cardioid-shaped trajectory, and the above process can be expressed by the Equation (7).

(6) $$F=\left\{\begin{array}{c} 1\\ -1 \end{array}\right.\begin{array}{cc} & \;\mathrm{if}\;r_{6}\leq 0.5\\ & \;\mathrm{else} \end{array}.$$

Here, F governs search directionality: F=−1 indicates positional values below the target reference ${(x}_{prey}>x_{new})$, while F=1 corresponds to values exceeding it ${(x}_{prey}<x_{new})$.

(7) $$x_{\mathrm{new}\;}=x_{\mathrm{prey}\;}+F\times \beta \times I\times x_{\mathrm{prey}\;}+F\times r_{3}\times \alpha \times d_{i}\times |cos(2\pi r_{4})\times [1-cos(2\pi r_{5})]|.$$

β is a constant, and the experiments in the original paper concluded that optimisation is best achieved when its value is taken as 6.

#### 2.1.5. Honey Mode

Under the honey mode, honey badgers are guided by honey guiding birds to quickly reach the vicinity of a target, at which point the individual honey badger updates its position using Equation (8). When the random number r ∈ (0,1) is less than 0.5, the honey badger was in digging mode, and when r was greater than 0.5, the honey badger was in honey mode.

(8) $$x_{\mathrm{new}\;}=x_{\mathrm{prey}\;}+F\times r_{7}\times \alpha \times d_{i}.$$

### 2.2. Set of Enhancement Strategies

In order to strengthen the optimisation capability of HBA, this paper adopts five optimisation strategies, and this section describes the principles of the five optimisation strategies and provides the implementation steps and implementation formulas.

- Good Point Set-based Population Initialization Strategy.

The essence of a good point set is to construct a uniformly distributed set of points within the s-dimensional unit cube. This set serves as a substitute for random numbers in Monte Carlo methods. Let $\gamma =(\gamma_{1},\cdots ,\gamma )\in C^{s}$, If the set of points formed by the first n terms of the collection $\left\{(\{\gamma_{1}k\},\cdots ,\{\gamma_{2}k\}),k=1,2,\cdots \right\}$ denoted $P_{n}$, satisfies the discrepancy bound:

(9) $$D(n,P_{n})\leq c(\gamma ,\varepsilon )n^{-1+\varepsilon },n=1,2,\cdots ,$$

then this collection is termed a good point set, and γ is called a good point. In practical applications, the following good points are recommended [18]:

(1) Square root sequence method

(10) $$\gamma =(\sqrt{p_{1}},\cdots ,\sqrt{p_{s}}).$$

(2) Prime number representation

(11) $$\gamma =(q,q^{2},\cdots ,q^{2}).$$

(3) Cyclotomic field method

(12) $$\gamma =(\gamma_{1},\gamma_{2},\cdots ,\gamma_{D})=(\{2cos\frac{2\pi }{p}\},\{2cos\frac{4\pi }{p}\},\cdots ,\{2cos\frac{2s\pi }{p}\}).$$

where p denotes a prime number satisfying p>2s+3, with s representing the solution dimensionality, and $q=p^{\frac{1}{s+1}}$. For practical implementation, solution bounds must be considered, hence solutions within the domain are obtained using the Equation (13), where $x_{j}^{max}$ and $x_{j}^{max}$ denote the upper and lower bounds of the solution respectively, i indicates the individual index, and j the dimension index.

(13) $$x_{i,j}=x_{j}^{min}+\{\gamma_{j}\cdot i\}\cdot (x_{j}^{max}-x_{j}^{min})(j=1,2,\cdots ,D).$$

- Decentralised Boundary Constraint Handling Strategy

The distributed boundary-handling strategy employs the Equation (14) to relocate out-of-bound individuals to distinct positions within feasible domains.

(14) $$X_{i,j}=\left\{\begin{array}{c} lb+rr\times \left(ub-lb\right)\;,\;\;X_{i,j}>ub\;or\;X_{i,j}<lb\;\\ X_{i,j}\;,\;\;lb<X_{i,j}<ub \end{array}\right..$$

- Sinusoidal function-based nonlinear convergence factor

The convergence factor balances exploration and exploitation capabilities within the algorithm. Building upon the original convergence factor, this paper proposes a sine-based nonlinear convergence factor implemented through the following formula:

(15) alpha=sin(((t/tmax)^c)∗π+π/2)+1,

where t denotes the current number of iterations, $t_{max}$ denotes the maximum number of iterations, c is a random number used to increase the randomness of the algorithm.

- Differential Evolution Strategy

Differential variation strategies are the core components of differential evolution algorithms for generating new candidate solutions to explore the space of optimisation problems, and differential evolutionary algorithm includes a variety of variation strategies, common strategies are DE/rand/1, DE/best/1, DE/current-to-best/1, DE/rand/2 [19], each method has its best use scenario and advantages and disadvantages. In this paper, we choose DE/current-to-best/1 to improve HBA, and the optimisation steps are as follows:

Step 1: In each iteration, the updated honey badger individuals are updated using the Equation (16), let the number of individuals in the population be N, the dimension be D, i∈(1,N), j∈(1,D), t be the number of the current iteration, m is a random number between 0 and 1.

(16) $$v_{i,j}(t+1)=x_{i,j}(t)+m\times (x_{best}-x_{i,j})+m\times (x_{r1,j}(t)-x_{r2,j}(t)).$$

Step 2: The crossover operation was performed using the Equation (17), with CR as the variance trigger determinant and rand as a random number between 0 and 1; the crossover operation reduces unnecessary computational overheads while being able to maintain population diversity.

(17) $$u_{i,j}(t+1)<\{\begin{array}{cc} v_{i,j}(t+1) & \;\mathrm{if}\;\mathrm{rand}\;<CR\\ x_{i,j}(t) & \;\mathrm{otherwise}\; \end{array}.$$

Step 3: The Equation (18) is applied to select the updated individuals, which retains the solution with the better fitness value between the original solution and the differentially mutated solution.

(18) $${\overset{\to }{X}}_{i}(t+1)=\{\begin{array}{c} {\overset{\to }{U}}_{i}(t)\;if\;f({\overset{\to }{U}}_{i}(t))\leq f({\overset{\to }{X}}_{i}(t))\\ {\overset{\to }{X}}_{i}(t)\;if\;f({\overset{\to }{X}}_{i}(t))\leq f({\overset{\to }{U}}_{i}(t)) \end{array}.$$

Although differential variation and genetic algorithms are similar in that they both have crossover and selection operations, DE uses the vectorial differences of individuals in the population for perturbation, which is essentially a guided stochastic search, whereas the crossover in GA is a structural reorganisation, and therefore, MCEHBA uses DE, but not a genetic algorithm.

- Gravity-Centred Opposition-Based Learning Strategy

Song et al. in 2020, for the limitations of the moth-flame optimisation algorithm (MFO), proposed the Gravity-Centred Opposition-Based Learning (GCOBL) strategy [20]. As an improvement strategy proposed in the field of intelligent optimisation algorithms in recent years, it is mainly applied to group intelligence algorithms, the following are the steps of this improvement strategy:

Step 1: Suppose there are N individuals in the population with dimension D. Each individual in the population is represented as $x_{i}=[x_{1},x_{2},......,x_{D}]$, a virtual centroid point, which indicates a potential optimal region in the current search space, is computed by taking the weighted average of all individual positions. The centroid calculation is formulated as:

(19) $$X_{\mathrm{gravity}\;}=\frac{1}{N}\sum_{i=1}^{N}X_{i}.$$

Step 2: Generate the inverse solution of the centre of gravity of the current solution to expand the search range to avoid falling into local optimum, the formula of the inverse solution is as follows:

(20) $$x_{op_{-}i}=2\times k\times X_{\mathrm{gravity}\;}-x_{i},$$

where k is the shrinkage factor, a random number between (0,1), and the solution with better fitness is selected among the original and centre of gravity reversal solutions into the next generation.

## 3. Algorithm Design and Structure

Within this section, we elucidate the rationale for adopting the enhancement strategies introduced in Section 2.2 and provide the algorithm pseudocode in Section 3.6.

### 3.1. Good Point Set-Based Population Initialization Strategy

In the absence of a priori knowledge, the individuals of the initialised population should be distributed as evenly as possible in the solution space. HBA generates the initial positions of the honey badger population through stochastic randomization. However, this approach exhibits three critical limitations: excessive randomness in the initial population leads to uneven distribution across the solution space; insufficient diversity diminishes the algorithm’s global exploration capability; low utilization of the search space increases susceptibility to local optima. To address these issues, this study introduces a good point set based population initialization strategy to optimize the initial phase of HBA. To validate the distribution advantages of good point set initialisation, this experiment employs a comparative approach, where initial populations of identical size are generated through both good point sets and random methods, with four metrics quantitatively evaluated: greater average nearest-neighbour distance indicates superior distribution uniformity, reduced distance standard deviation reflects enhanced inter-individual spacing consistency; an increased minimum inter-individual distance signifies diminished clustering intensity; while a spatial coverage ratio approaching the numerical value 1 indicates enhanced regional coverage. Experimental results across three distinct dimensions (Table 1) consistently demonstrate that good point set initialisation significantly outperforms random initialisation across all four metrics—manifesting as increased average nearest-neighbour distance, decreased distance standard deviation, elevated minimum inter-individual distance, and spatial coverage ratio converging towards unity—thereby confirming its efficacy in enhancing spatial distribution uniformity and regional coverage completeness within initial populations.

### 3.2. Decentralised Boundary Constraint Handling Strategy

In HBA, newly generated solutions may exceed predefined boundaries after individual position updates, Table 2 records the number of times an individual crossed the boundary in five runs of HBA. For each run, the population size was set to 30, the solution vector dimension to 30, and the maximum iterations to 1000. Since each iteration generates a new set of individuals, the average number of out-of-bound solutions per iteration and the maximum out-of-bound count per iteration were recorded for all runs.

The experimental results reveal that, among 1000 solution vectors generated per iteration, an average of 3 vectors violated boundaries, while the maximum number of violations exceeded 200. This indicates that although the average number of times the solution vectors cross the boundary is not large, there is always a probability that 20% of the solution vectors will exceed the boundary. However, the strategy of HBA is to transfer all individuals larger than the upper boundary to the upper boundary, i.e., make their values equal to ub, and to transfer all individuals smaller than the lower boundary to the lower boundary, i.e., to make their values equal to lb. This practice can solve the problem of individual solution vectors exceeding the boundaries, but it will make a large number of solution vectors equal to the same value, which will lead to the convergence of the individuals, and the poor diversity of populations, so in this paper, we adopt the decentralised boundary constraint handling strategy: for each individual that exceeds the boundary, it is transferred to a different location within the boundary using the Equation (14), which effectively enhances the population diversity and improves the probability of finding the optimal solution.

### 3.3. Nonlinear Convergence Factors Based on Sinusoidal Functions

**Figure 1:**
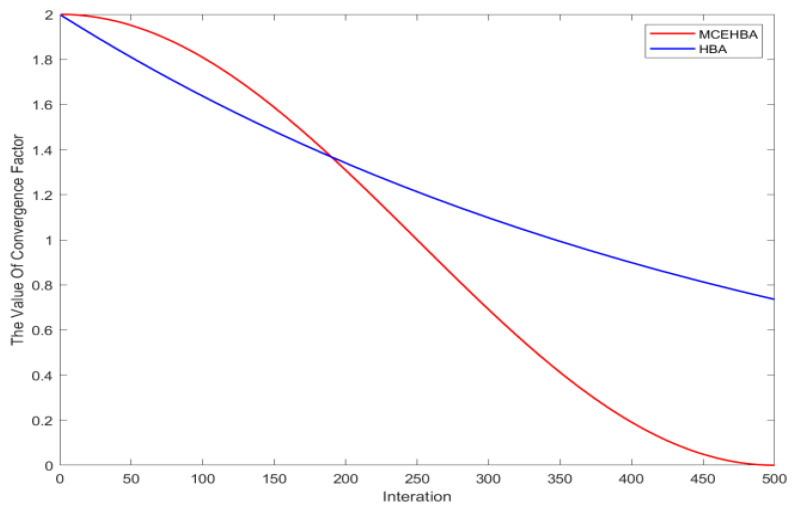
Schematic of nonlinear convergence curves based on sinusoidal functions.

The nonlinear convergence factor is used to control the transition of an individual from exploration to exploitation. The convergence factor of HBA has obvious limitations in regulating the switch between global exploration and local exploitation modes, and its parameter change slope varies very little, which leads to the algorithm not being able to dynamically adapt to the needs of the complex search environment during the iterative process and the minimum value of the convergence factor is not reset to zero, making the local search phase lack of full convergence ability, easy to fall into sub-optimal solution. To address this problem, this paper proposes a nonlinear convergence factor improvement strategy based on the sinusoidal function, and reconstructs the parameter update mechanism by introducing the nonlinear characteristics of the sinusoidal function. the convergence curve is shown in Figure 1 During the initial iterations, the convergence factor maintains a high value to enhance global exploration capability. As iterations progress, its decay rate exhibits nonlinear acceleration. In later stages, the factor stabilizes at a low value. Unlike HBA, MCEHBA’s convergence factor strictly decreases to zero upon termination, significantly improving local exploitation precision in the final phase.

### 3.4. Differential Evolution Strategy

DE/current-to-best/1 can deal with the problem of premature convergence of the honey badger algorithm by introducing the difference between the current individual and the optimal individual as well as the difference of the random individual, i.e., guiding the individual to move to a better area, and maintaining the diversity of the population through the random perturbation.

### 3.5. Gravity-Centred Opposition-Based Learning Strategy

The Gravity-Centred Opposition-Based Learning Strategy combines the theory of centre of gravity and the reverse learning mechanism, and enhances the development of directionality while maintaining the exploration capability of the algorithm, which can significantly improve the convergence speed and accuracy. In order to solve the problem that the honey badger optimisation algorithm is prone to fall into the problem of local optimum, in this paper, we use the GCOBL to improve the diversity of honey badger’s population, and the dynamic adjust the search direction.

### 3.6. Time Complexity Analysis and Pseudocode

Given a population size N, dimensionality D, and maximum iterations T, the computational costs for MCEHBA are quantified as follows: good point set initialisation requires ND operations, distributed boundary-handling requires one operation, the sine-based nonlinear convergence factor requires one operation, differential mutation D, and centroid opposition-based learning ND, Algorithmic structure analysis yields a total running time of ND + 3 × NDT with asymptotic time complexity O(ND + NDT); while conventional HBA exhibits ND + NDT operations and identical asymptotic complexity O(ND + NDT). The pseudocode of MCEHBA is presented in Algorithm 1, where the improvement strategies proposed in this work are highlighted in bold.
**Algorithm 1.** The pseudocode of MCEHBAStart MCEHBA; Input: Set the population parameter size N, the population dimension D, the maximun number of iterations Max, upper and lower bounds ub,lb **The initial population *X*** is generated via good point set initialisation (Equations (12) and (13)); Evaluate the fitness values of the initial population and designate the optimal value as $X_{prey}$; While t < Max do;  **Update the convergence factor using Equation (15);**  Update odour concentration using Equation (2);  For I = 1: N do;    Update the value of F using Equation (6);     For j = 1:D do;      If r < 0.5        Update the position of the individual using Equation (7)      Else        Update the position of the individual using Equation (8);      End    End    **Using Equation (14) for out-of-bounds processing;**    Evaluating the adaptation value of the new individual, if the adaptation value is better than the corresponding individual in X, the new individual overwrites the original individual;    **Individuals were differentially varied using the Equations (16)–(18);**    Evaluating the adaptation value of the new individual, if the adaptation value is better than the corresponding individual in X, the new individual overwrites the original individual;  End  **Using Equation (14) for out-of-bounds processing;**  Evaluate adaptation values for new populations and update $X_{prey}$;  **Perform the Gravity-Centred Opposition-Based Learning Strategy using the Equations (19) and (20)**  Evaluate the new fitness value of the population, keeping the current optimal solution vector with the optimal fitness value End Output: Solution vectors and optimisation results. End MCEHBA

## 4. Algorithm Experimentation and Testing

### 4.1. Test Functions and Parameter Settings

In order to accurately test the performance of MCEHBA, as well as to analyse the feasibility of the GCOBL strategy under the population variation strategy based on differential evolution strategy, this paper uses a set of cec2017 test functions for comparative analysis of MCEHBA, including 29 functions (F1, F3~F30), covering four types of typical optimisation problems: F1 and F3 are single-peak functions, which are convex functions constructing globally unique optimal solutions based on quadratic and linear offsets, used to test the efficiency of the algorithm’s local development; F4–F10 are multi-peak functions integrating oscillatory functions with exponential local extremes, verifying the global search capability through a multimodal landscape; F11–F20 are hybrid functions, weighted superposition of 12 basis functions by nonlinear transformation to generate discontinuous and asymmetric high-dimensional coupling space; F21–F30 are compositional functions, Gaussian noise perturbation and heterogeneous parameter configurations are introduced on the basis of the hybrid functions, and complex interference environment is simulated by noise injection and heterogeneous operation. In the experiment, the number of iterations is set to 500, the population size is 50, the variance departure determinant CR in Equation (17) is set to 0.5, and the constant c in Equation (15) is set to 1.

### 4.2. Analysis of the Effectiveness of Optimization Strategies

In order to reflect the independent and combined optimisation effects of various optimisation strategies in MCEHBA, this paper conducts ablation experiments on MCEHBA, using HBA with five variants of HBA,, in which HBA_Variant1 adopts a sinusoidal function-based nonlinear con-vergence factor on the basis of HBA; HBA_Variant2 adopts the good point set initialisation strategy on the basis of HBA_Variant1; HBA_Variant3 adopts the decentralised transgression processing strategy on the basis of HBA_Variant2, HBA_Variant4 adopts the differential evolution strategy on the basis of HBA_Variant3, HBA_Variant5 adopts the GCOBL strategy on the basis of HBA_Variant4, and to comprehensively evaluate the performance of MCEHBA across varying problem dimensionalities, this section conducts experiments under two configurations: 30-dimensional and 50-dimensional search spaces.in each experiment the four algorithms are run independently for 30 times, and get the average adaptation value, the variance of the adaptation value, the maximum value of the adaptation value and the minimum value of the adaptation value, These metrics collectively reflect algorithmic performance across four dimensions: overall efficacy, stability, solution potential, and risk mitigation capability. Table 3 and Table 4 show the frequency of algorithms with this strategy that are better than the control group in terms of adaptation value (For example, the frequency of HBA Variant2 is better than that of HBA Variant1), see Appendix A for complete experimental data.

Experimental data from HBA Variant1 and HBA Variant2 reveal fundamental principles of algorithmic improvement: there exists no universal strategy, only context-adaptive optimization. The sinusoidal-based nonlinear convergence factor enhanced convergence efficiency in 57% of non-convex functions, validating its regulatory value in multi-peak scenarios. The optimal point set demonstrated superior performance in 50% of mixed and combinatorial functions, confirming its sensitivity to solution space structure. Although the optimal point set showed no significant advantage across 29 functions, it remains irreplaceable in specific problem subcategories. Therefore, in practical engineering applications, this study’s experimental conclusions enable flexible algorithm adjustments—determining whether to employ the optimal point set initialization strategy or the sinusoidal-based nonlinear convergence factor strategy based on problem type.

It is worth noting that in terms of the average value, HBA Variant5 outperforms HBA Variant4 20 times, HBA Variant4 outperforms HBA Variant3 25 times, and HBA Variant3 outperforms HBA Variant2 20 times, This strongly proves that the use of decentralized overstepping processing strategy, differential variation strategy and GCOBL can improve the performance of the algorithm. Also, on test functions F6 and F11, the fitness of each variantare gradually decreasing, which fully illustrates the progressive positive optimisation effect of the nonlinear convergence factors combining good point set initialisation with decentralised transgression processing strategies, differential evolution strategy and centre of gravity reverse learning strategy on the HBA algorithm.

To further verify the performance of MCEHBA, this paper increases the problem dimension to 50, repeats the above experiments and the experimental results are shown in Table 4.

At 50 dimensions, HBA Variant 1 demonstrates superior fitness values over HBA in only 7 test functions, indicating that the sine-based nonlinear convergence factor experiences compromised efficacy in high-dimensional problems. Consequently, for engineering applications involving high-dimensional optimisation, reverting to the original convergence factor may be considered. Notwithstanding this limitation, the implementation of multiple enhancement strategies in MCEHBA maintains statistically significant performance advantages over conventional HBA across all environments. Each variant demonstrates progressively increasing superiority over its control group, with HBA Variant 4 outperforming HBA Variant 3 in 26 test functions. In the 50-dimensional case, HBA Variant 1 demonstrated better fitness values than HBA in only 7 test functions. This indicates that the efficacy of the sine-based nonlinear convergence factor is affected in high-dimensional problems. However, HBA Variant 1 achieved superior results on 5 out of 10 composite functions (F21–F30). Therefore, for engineering applications involving high-dimensional optimization where the objective function is not composite, reverting to the original convergence factor could be considered.

Despite this limitation, after implementing multiple enhancement strategies in MCEHBA, it maintains a significant performance advantage over the traditional HBA across all tested environments. The superiority of each variant progressively increases compared to the control group. Notably, HBA Variant 4 outperformed HBA Variant 3 on 26 test functions.

On the multi-peak functions from F4 to F10, MCEHBA achieved superior performance, securing optimal results on 4 benchmark functions and outperforming HBA in 6 out of 7 test instances. The results show that the fusion strategy adopted in this paper can significantly improve the global search ability.

### 4.3. Comparison with Other Excellent Swarm Intelligence Algorithms

In order to fully reflect the excellent ability of MCEHBA, in addition to HBA, five excellent algorithms: DOA [21], GWO [22], HHO [23], MFO [24], SO [25] and WOA are selected for comparison with it in this section, testing 7 swarm intelligence optimisation algorithms using the CEC2017 test function, take the dimension is equal to 50, each algorithm independently run for 30 times, and get the average adaptive value, the variance of the adaptive value, the maximum value of the adaptive value and the minimum value of the adaptive value, its results are displayed in Table 5.

When compared with other excellent algorithms, MCEHBA obtained all the single-peak functions, indicating that its local search capability is superior to other algorithms. However, the global search capability, although improved over HBA, is still prone to local optimal solutions compared to other algorithms on the multi-peak benchmark function from F4 to F10. In the hybrid function from F11 to F20 and the compositional function from F21 to F30, MCEHBA only loses four of the benchmark functions, indicating that MCEHBA is able to handle most of the complex problems with high quality compared with other excellent algorithms.

We found that MCEHBA show large standard deviations, this variability does indeed raise valid questions about robustness and highlights a fundamental challenge in algorithm design.

Our perspective aligns with the insight that there is no universally optimal algorithm for all types of optimization problems. The large standard deviations observed often stem from several key factors intrinsic to complex, real-world-inspired problems like those in CEC2017, including Algorithm Stochasticity, Sensitivity to Parameters and Initialization.

It is worth noting that in the performance comparison experiments with other good swarm intelligence optimisation algorithms, the variance of DOA is optimal on 3 functions, that of GWO is optimal on 1 function, that of SO is optimal on 10 functions, and the other two algorithms are optimal on 0 functions. Therefore, although MCEHBA only performs best in 15 functions, its robustness is still better than the other six algorithms in comparison.

### 4.4. Convergence Analysis

**Figure 2:**
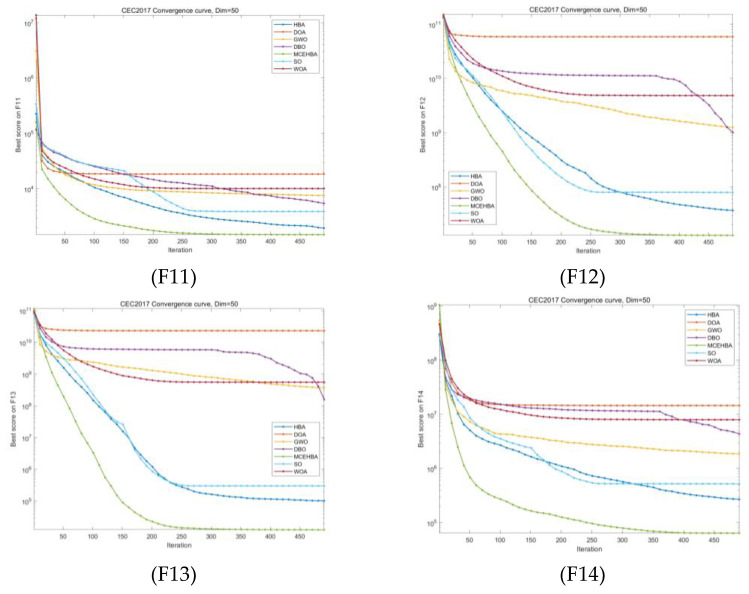

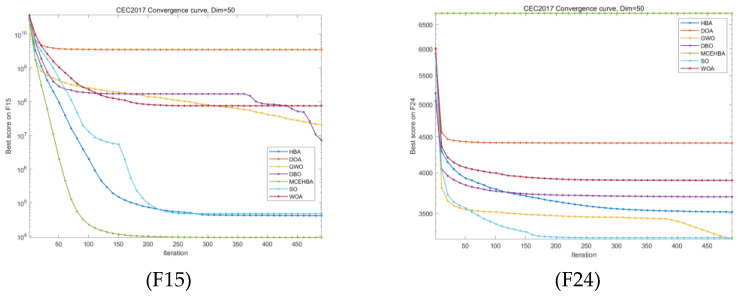
Partial convergence diagram.

Comparison experiments based on the CEC2017 benchmark test set show that MCEHBA exhibits significant advantages in convergence characteristics and robustness. The convergence curve comparison results (see Figure 2) show that MCEHBA significantly outperforms the comparison algorithms in terms of convergence accuracy on 20 benchmark functions, in the benchmark functions F11–F15 and other functions, MCEHBA is able to converge quickly in the early iteration period, which verifies the fast response of the algorithm in complex nonlinear problems; it is worth noting that, in the functions F13, F14, etc., which contain the noise interference and dimensional coupling, the convergence trajectory of MCEHBA consistently exhibits a monotonically decreasing characteristic, which proves that its population updating strategy that combines the differential evolution strategy with the GCOBL can effectively maintain the stability of the search direction. However, MCEHBA exhibits limitations in handling specific optimization challenges, it failed to converge on the F24 benchmark function, primarily due to its inability to effectively counteract misguided gradient directions caused by strong interdimensional coupling in highly rotated non-separable problems, root cause analysis reveals that the current mutation strategy inadequately adapts to the rotational transformation matrix. Therefore, on similar problems, MCEHBA needs to be further improved to enhance the universality.

### 4.5. Feldman Test

To demonstrate the rigour of the results in this section the results in are subjected to the Friedman test. The Friedman test is a non-parametric statistical method for comparing the differences between three or more correlated groups. In the context of significance testing of intelligent optimisation algorithms, the Friedman test can often be used to assess the performance of a wide range of algorithms on multiple benchmark problems, by performing a Friedman test to obtain the average rank of each algorithm, the smaller the average rank, the better the performance of the algorithm.

MCEHBA has significant difference and best results compared to other algorithms, Table 6 gives seven excellent algorithms with their average rank, in which the average rank of MCEHBA is the smallest among the seven algorithms, which indicates that MCEHBA has significant difference and best results compared to other algorithms.

**Figure 3:**
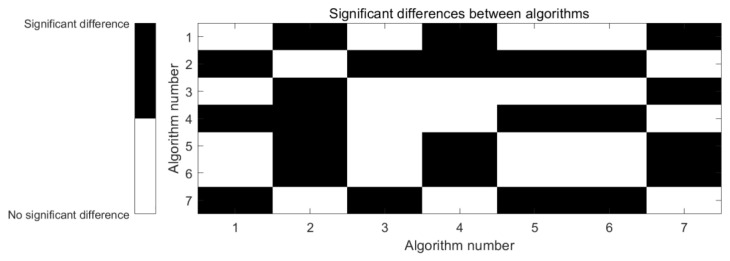
Statistical significance heatmap.

The Friedman test yielded a *p*-value < 0.05, indicating statistically significant performance differences among the algorithms across 29 CEC test functions (α = 0.05). To identify specific difference sources, we conducted a Nemenyi post-hoc test with a critical difference (CD) of 1.6724. Figure 3 presents a heatmap of differences based on mean rankings, where Algorithm number 1–7 correspond to: 1-HBA, 2-DOA, 3-GWO, 4-DBO, 5-MCEHBA, 6-SO, 7-WOA.

The results in Figure 3 show MCEHBA’s significant superiority over DOA, DBO and WOA. No statistically significant differences exist between MCEHBA and HBA, GWO or SO. Notably, MCEHBA achieved significantly lower average rankings than HBA, GWO and SO. When considered alongside its attainment of optimum solutions on 20/29 test functions, this indicates superior overall optimization potential.

### 4.6. Engineering Applications

Engineering optimisation problems challenge the global search capability and convergence accuracy of intelligent algorithms due to their nonlinear constraints, multi-peak characteristics and high-dimensional complexity, MCEHBA enhances the algorithm’s exploration-exploitation capabilities in complex engineering search spaces by integrating the differential evolution strategy, the GCOBL mechanism and the nonlinear convergence factors combining good point set initialisation with decentralised transgression processing strategies. In order to systematically verify the engineering usability of MCEHBA, three types of typical engineering optimisation problems are selected for the study: welded beam design problem, reducer design problem and cantilever beam design problem.

(1) Welding beam design problem

The goal of welded beam design is to determine the optimal geometric parameters that make it possible to fabricate the beam at the lowest cost while ensuring that it meets the constraints of shear stress, bending stress, and deflection when subjected to loads, possessing four design variables: the height of the weld $x_{1}$, the length of the weld $x_{2}$, the height of the beam $x_{3}$, and the width of the beam $x_{4}$.

The overall objective function is equal to the sum of the weld material cost and the beam material cost with the following Equation (21):

(21) $$\mathrm{Cost}\;=1.10471{x_{1}}^{2}x_{2}+0.04811x_{3}x_{4}(14+x_{2})$$

Various types of realistic constraints need to be observed in solving the welded beam design problem, including shear stress constraints (g1), bending stress constraints (g2), deflection constraints (g3), geometrical constraints (g4), flexural stability constraints (g5), lower limit of the weld height constraints (g6), and upper limit of the cost constraints (g7), and the constraints are formulated as follows, where the value of P is taken to be 6000, the value of L is taken to be 14, E is taken as 30,000,000 and G is taken as 12,000,000.

(22) $$M=P(L+0.5x_{2}),$$

(23) $$R=\sqrt{\frac{x_{2}^{2}}{4}+{\left(\frac{x_{1}+x_{3}}{2}\right)}^{2}},$$

(24) $$g1\;=\sqrt{{\left(\frac{P}{\sqrt{2}x_{1}x_{2}}\right)}^{2}+2\cdot \frac{P}{\sqrt{2}x_{1}x_{2}}\cdot \frac{MR}{J}\cdot \frac{x_{2}}{2R}+{\left(\frac{MR}{J}\right)}^{2}}\leq \tau_{max}=13600,$$

(25) $$g2=\frac{6PL}{x_{4}x_{3}^{2}}\leq \sigma_{max}=30000psi,$$

(26) $$g3=\frac{4PL^{3}}{Ex_{4}x_{3}^{3}}\leq \delta_{max}=0.25,$$

(27) $$g4=x_{1}-x_{4}\leq 0,$$

(28) $$g5=\frac{4.013E\sqrt{\frac{x_{3}^{2}x_{4}^{6}}{36}}}{L^{2}}(1-\frac{x_{3}}{2L}\sqrt{\frac{E}{4G}}),$$

(29) $$g6=0.125-x_{1}\leq 0,$$

(30) $$g7=1.10471x_{1}^{2}x_{2}+0.04811x_{3}x_{4}\left(14+x_{2}\right)\leq 5.$$

Combining the above constraints with the objective function can get the results as in Table 7, where the use of MCEHBA algorithm presents optimal results that can lead to the lowest cost of manufacturing beams.

(2) Reducer design problem

Reducer design is a classical optimisation problem in mechanical engineering, aiming to minimise the volume of the reducer by adjusting key parameters such as gears and shafts, while satisfying strength, transmission efficiency and geometric constraints. There exist seven key design parameters: $x_{1}\;$ represents the tooth width, $x_{2}$ represents the module number, $x_{3}$ represents the number of pinion teeth, $x_{4}$ represents the diameter of the shaft 1, $x_{5}$ represents the diameter of the shaft 2, $x_{6}$ represents the length of the input shaft, and $x_{7}$ represents the length of the output shaft.

The total objective function includes gear volume and shaft volume with the following Equation (31):

(31) $$0.7854x_{1}x_{2}^{2}(3.3333x_{3}^{2}+14.9334x_{3}-43.0934)+7.4777(x_{6}^{3}+x_{7}^{3})+0.7854(x_{4}x_{6}^{2}+x_{5}x_{7}^{2}).$$

Also, the design of the reducer needs to conform to various types of realistic constraints, including stress constraints (g1~g4), shaft strength constraints (g5,g6) and geometric constraints (g7~g11).

(32) $$g1=x_{1}x_{2}^{2}x_{3}\geq 27,$$

(33) $${g2=x}_{1}x_{2}^{2}x_{3}^{2}\geq 397.5,$$

(34) $$g3=\frac{x_{4}^{3}}{x_{2}x_{3}x_{6}^{4}}\leq \frac{1}{1.93},$$

(35) $$g4=\frac{x_{5}^{3}}{x_{2}x_{3}x_{7}^{4}}\leq \frac{1}{1.93},$$

(36) $$g5=\frac{1}{110x_{6}^{3}}\sqrt{{\left(\frac{745x_{4}}{x_{2}x_{3}}\right)}^{2}+16.9\times 10^{6}}-1\leq 0,$$

(37) $$g6=\frac{1}{85x_{7}^{3}}\sqrt{{\left(\frac{745x_{5}}{x_{2}x_{3}}\right)}^{2}+157.5\times 10^{6}}-1\leq 0,$$

(38) $${g7=x}_{2}x_{3}\leq 40,$$

(39) $$g8=x_{1}\geq 5x_{2},$$

(40) $$g9=x_{1}\leq 12x_{2},$$

(41) $$g10=x_{4}\geq 1.5x_{6}+1.9,$$

(42) $$g11=x_{5}\geq 1.1x_{7}+1.9.$$

Combining the above constraints with the objective function results can be obtained as shown in Table 8, where the use of MCEHBA presents better results and is able to design the reducer with the smallest volume.

(3) Cantilever Beam Design Problem

A cantilever beam is a beam structure that is fixed at one end and free at the other, and is widely used in machinery and construction. The optimisation objective is to minimise the material usage while satisfying the stiffness (deformation) constraints, and the design variables are the section heights of each segment of the cantilever beam arm $x_{1}\sim x_{5}$, the objective function is the volume of the total material, and the Equation (43) is as follows:

(43) $$f=0.0624\sum_{i=1}^{5}x_{i}.$$

Solving the cantilever beam design problem requires satisfying stiffness constraints to ensure that the free end deflection does not exceed the permissible value, as given in the following Equation (44):

(44) $$g1=\frac{61}{x_{1}^{3}}+\frac{37}{x_{2}^{3}}+\frac{19}{x_{3}^{3}}+\frac{7}{x_{4}^{3}}+\frac{1}{x_{5}^{3}}-1\leq 0.$$

Combining the above constraints with the objective function results can be obtained as shown in Table 9, where the use of MCEHBA presents better results, being able to minimise the volume of the total material.

## 5. Mobile Robot Path Planning Based on Grid Method

### 5.1. Procedure

#### 5.1.1. Generate Obstacle Field

Let the maximum diameter of the robot in the two-dimensional plane projection is d. To ensure the accuracy of the path planning results and at the same time to improve the efficiency of path planning, the width of the grid is taken to be d, and the robot can be represented by a grid.

**Figure 4:**
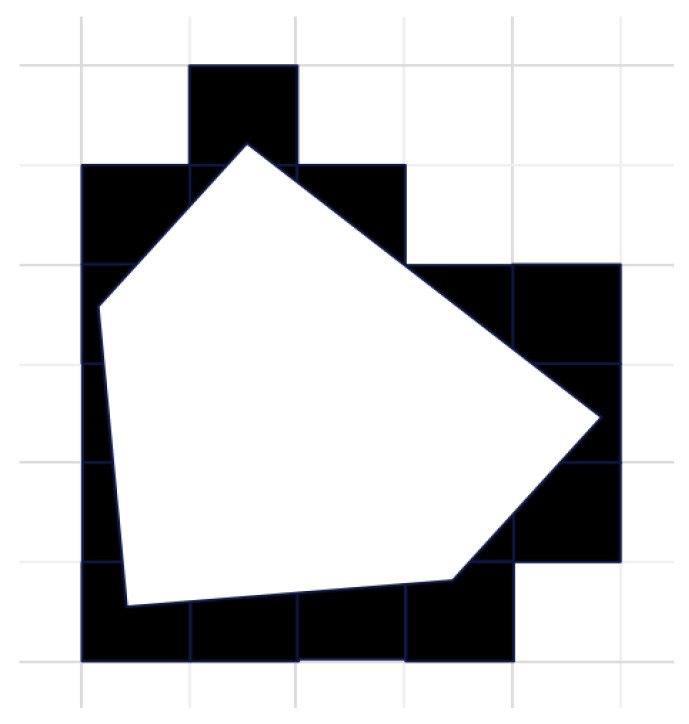
Obstacles in the grid (White represents real-life irregular obstacles, black represents inflated regular obstacles).

The obstacle field is a mapping of the collection of obstacles in the real farmland environment, firstly, the actual obstacle contours are mapped to the grid matrix by top view projection, at this time, obstacle areas are irregular shapes or polygons (as shown in the light-coloured part of Figure 4). To establish a computationally tractable set model, this study employs an obstacle inflation strategy to expand safety boundaries around obstacles for collision avoidance, specifically, to ensure collision-free navigation of the robot, any grid cell that has spatial intersection with the original obstacle is marked as a forbidden area (as shown in the black part of Figure 4), all obstacles in the farmland environment can be processed according to this method to obtain the obstacle field, and can be expressed using the Cartesian coordinate system, in which the centre coordinates ($x_{i},y_{i}$) of the grid cells are calculated as: $x_{i}=i\times d+d/2,y_{i}=j\times d+d/2$, defining the set consisting of all obstacle coordinates as B.

#### 5.1.2. Design of Objective Function Functions and Constraints

Establish a planar rectangular coordinate system A on the obstacle field and specify the lower left corner as the origin, and let the grid map have n×n grids.

In the Honey Badger optimisation algorithm, each population consists of n individuals, where each individual represents a potential path solution. On this basis, the set of solutions generated by a population is defined as an ordered array $X0=\left[x1,x2\ldots \ldots xn\right]$, given that the path must be connected from the coordinates $\left(1,1\right)$ to the coordinates $\left(n,n\right)$, in order to ensure that the paths are coherent, it is necessary to identify a point that the robot is bound to pass through in each row of the obstacle field. Define the elements of array X as the x-coordinates of the mandatory path points, and set the y-coordinates as a sequential integer array from 1 to n, the coordinates of the mandatory points can then be derived as $\left(x1,1\right),\left(x2,2\right)\ldots (xn,n)$.

However, the security of the movement path represented by using only the mandatory points is not strong, the path points are too few, and two adjacent mandatory points are connected using a straight line, the probability of collision between the robot and the obstacle is extremely high, to ensure that the robot can only move in the eight field range, interpolation is performed on every two mandatory points to obtain a coherent path point, the interpolated array of path points is defined as $X=\left[x1,x2\ldots \ldots xm\right],\;m\geq n$.

The core task of mobile robot path planning is to seek the global optimal trajectory for the robot, and its optimisation objective needs to take into account a number of energy losses generated during the operation process. From the perspective of dynamics, the main sources of energy consumption can be divided into two dimensions: driving energy consumption and steering energy consumption, driving energy consumption is positively correlated with the path length, and its physical nature stems from the continuous output power of the power system and the friction resistance overcome during the movement; steering energy consumption is directly related to the steering frequency and steering amplitude, involving the mechanical wear of the transmission mechanism and the kinetic energy loss during the switching of the motion state. Therefore, A scientific objective function is constructed not only to reduce the basic energy consumption by minimising the path length, but also to quantify the additional cost of handling steering manoeuvres by introducing a steering cost function. Based on this the objective function is designed as follows:

(45) $$f=f_{1}+f_{2}+f_{3}$$

where $f_{1}$ is the distance cost function, $f_{2}$ is the turn cost function and $f_{3}$ is the safety cost function. $f_{1}$ is calculated as follows, the distance cost is equal to the sum of the Cartesian distances of the path points.

(46) $$f_{1}=\sqrt{{\left(x_{i}-x_{i+1}\right)}^{2}+{\left(y_{i}-y_{i+1}\right)}^{2}},(i<m-1)$$

Equation (49) is used to calculate the steering cost function, from the coordinates $p_{i}\left(x_{i},y_{i}\right),{\;p}_{i+1}\left(x_{i+1},y_{i+1}\right),{\;p}_{i+2}\left(x_{i+2},y_{i+2}\right)$ can be obtained as vectors $X=\overset{\to }{p_{i+1}p_{i}},Y=\overset{\to }{p_{i+1}p_{i+2}}$, where a denotes the product of the lengths of X and Y, b denotes the dot product of X and Y, and c denotes the vector consisting of X and Y composed of the angle of the cosine value, when the cosine value is larger, it means that the robot turns the greater the energy consumed, when c=−1 means that the robot goes straight, no turn energy consumption, when c=0 means that the robot needs to turn 90°, $f_{2}$ need to increase a certain penalty value, the penalty value in the Equation (50) to meet $w_{1}<w_{2}<w_{3}<w_{4}<w_{5}$.

(47) $$a=\sqrt{{\left(x_{i}-x_{i+1}\right)}^{2}+{\left(y_{i}-y_{i+1}\right)}^{2}}\times \sqrt{{\left(x_{i+2}-x_{i+1}\right)}^{2}+{\left(y_{i}-y_{i+1}\right)}^{2}}$$

(48) $$b=(x_{i}-x_{i+1})\times {(x}_{i+2}-x_{i+1})+(y_{i}-y_{i+1})\times {(y}_{i+2}-y_{i+1})$$

(49) c=b/a

(50) $$f_{2}=\{\begin{array}{c} w_{1},c=-1\\ w_{2},c=-\frac{\sqrt{2}}{2}\\ w_{3},c=0\\ w_{4},c=\frac{\sqrt{2}}{2}\\ w_{5},c=1 \end{array}$$

Equation (51) represents the safety cost of the path, which is $w_{6}$ when the path crosses an obstacle and satisfies $w_{6}\gg w_{5}$.

(51) $$f_{3}=\left\{\begin{array}{c} w_{6},(x_{i},y_{i})\in B\\ 0,(x_{i},y_{i})\notin B \end{array}\right.$$

#### 5.1.3. Improvement of Neighbourhood Search Strategies

**Figure 5:**
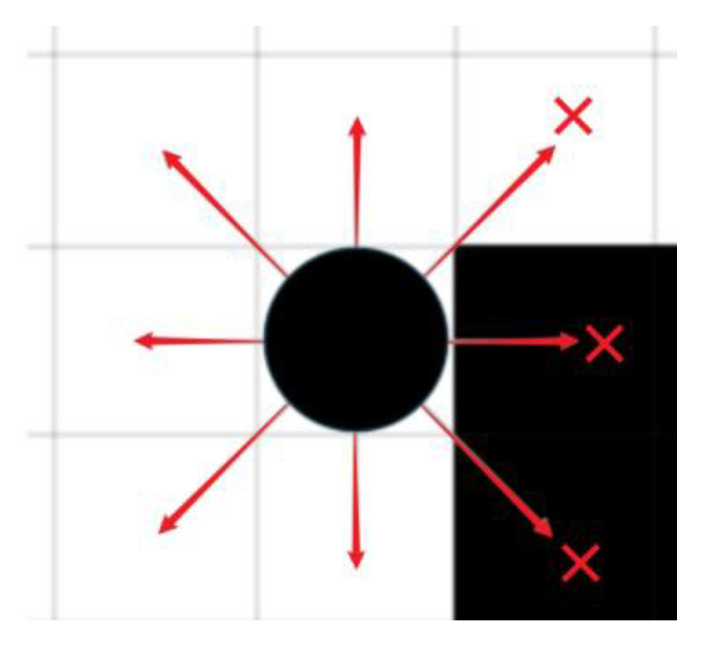
Robots search their fields. (black squares refer to obstacles, black circles refer to the robot, red arrows refer to the grid (direction) that the robot can choose in the next step, and red crosses indicate that this direction will collide with the obstacle).

**Figure 6:**
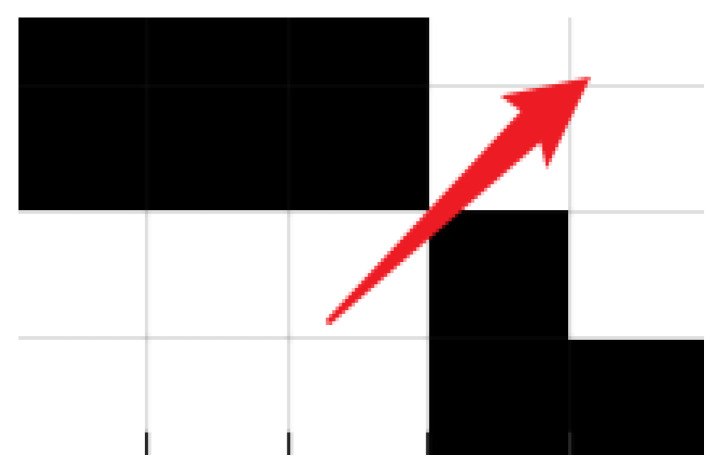
Robot passes through obstacles. (black parts represent obstacles, red arrows represent the direction of robot movement).

When performing path planning, the algorithm will sequentially search the eight neighbourhoods of its current location and evaluate their advantages and disadvantages, as shown in Figure 5, the black solid point in the figure indicates the current location of the robot, and the arrows indicate the direction of movement that the robot can choose, and the neighbourhoods to its right and lower right are obstacles, which will be excluded from searching; the neighbourhood in the upper right does not contain obstacles, but evaluated from the aspect of safety, since the robot itself has a certain volume, when moving along the diagonal direction of the grid may collide or friction with obstacles, as in Figure 5 or even pass through the obstacles as in Figure 6, so this paper adds improvements to the neighbourhood search aspect based on the initial search strategy, when the robot chooses the next step in the neighbourhood, if a certain direction, although it will not pass through the obstacles directly, may collide or friction with the obstacles, the algorithm will still regard this path as having $w_{6}$ safety cost and avoid this path, which effectively improves the safety of the mobile robot, the improved neighbourhood search strategy is formulated as follows, Equation (52) is the improved objective function.

(52) $$f_{4}=\left\{\begin{array}{c} w_{6},(x_{i},y_{i+1})\in B\;or\;(x_{i+1},y_{i})\in B\\ 0,(x_{i},y_{i+1})\notin B\;and\;(x_{i+1},y_{i})\notin B \end{array}\right.$$

(53) $$f=f_{1}+f_{2}+f_{3}+f_{4}$$

### 5.2. Program Setting and Parameter Setting

**Figure 7:**
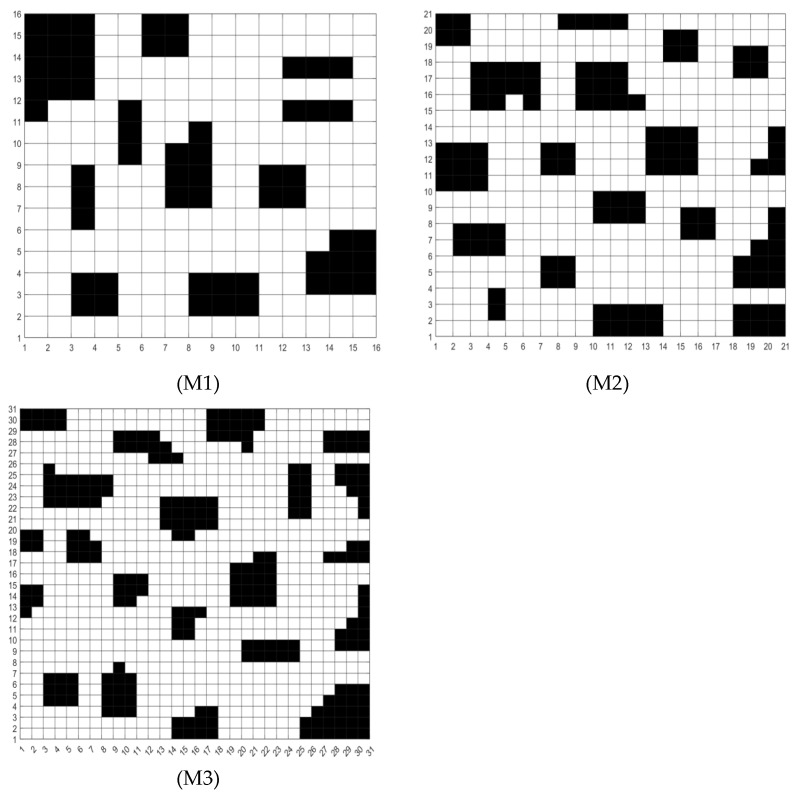
Grid map. (Black parts represent obstacles).

In order to verify the engineering applicability of MCEHBA for robot path planning in complex farmland scenarios, this paper designs three experimental scenarios with different path planning difficulties based on typical farmland operating environment features, each scenario corresponds to a grid map, and the information of the maps are shown in Table 10 and Figure 7 grid map, In the table, the map size is displayed as 20 × 20 grid units, while the actual physical dimensions will be scaled proportionally (e.g., 200 × 200 m). Furthermore, the obstacle density Op is calculated as follows:

(54) $$Op=\left(\frac{Num_{obstacles}}{Num_{total}}\right)\times 100\%,$$

where $Num_{obstacles}$ denotes the count of obstacle grids, $Num_{total}$ represents the total number of grids in the map, which can effectively simulate the challenges that exist in real farmland operations, such as traversing between ridges and avoiding irregular obstacles, in each experimental scenario, four intelligent optimisation algorithms: GWO, DOA, HBA, MCEHBA, are selected to run and compare their performances. For maps M1 and M2, the number of iterations is set to be 600; For map M3, because of the complexity of the map, the number of iterations is increased to be 1000, the parameter adjustment in the manuscript only involves one map, and there is no comparison between maps in the experiment, this ensures a direct and fair comparison of their path planning performance on the same problem instance. The population size N is 100, the dim is equal to the number of rows in the map. To avoid randomness in results, each algorithm was run 5 times on each map. The penalty values $w_{1},{\;w}_{2},w_{3},w_{4},{\;w}_{5},{\;w}_{6}$ in Equations (50) and (51) are taken as 0, 20, 40, 60, 80, 9,999,999 respectively.

### 5.3. Simulation Results and Analysis

In order to comprehensively test the path planning capability of MCEHBA, in this paper, we record the mean (Mean), variance (Std), worst (Worst) and optimum (Best) of each algorithm for five runs on three maps, give the roadmap and convergence diagram of each algorithm and analyse the experimental results.

#### 5.3.1. Analysis of Results for Map M1

According to the planning result data in Table 11, MCEHBA shows significant overall performance advantages in path planning, with the mean value of MCEHBA (140.95) being the lowest among all the algorithms, which is 23.1%, 45.1% and 11.0% lower than that of HBA, DOA and GWO, respectively, indicating that it performs the best on average in the goal optimisation task, its best (124.49) and worst (165.66) values are also better than the other algorithms, and although its standard deviation (508.53) is higher than that of GWO (358.93), it is much lower than that of HBA (1056.90) and DOA (3996.19), which suggests that its stability is still in the acceptable range.

**Figure 8:**
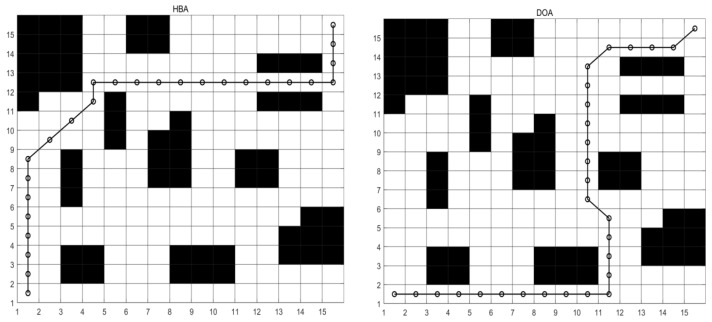

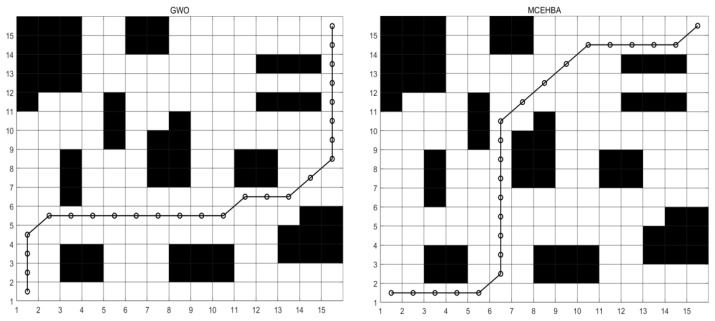
Route map of M1. (The black parts represent obstacles, and the black lines represent the motion paths planned by the robot).

**Figure 9:**
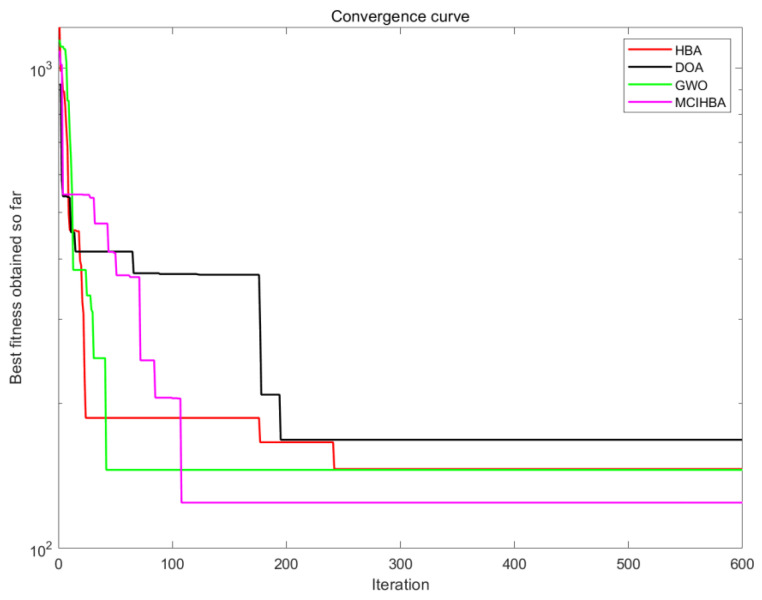
Convergence diagram of M1.

Figure 8 and Figure 9 show that the planned path of MCEHBA exhibits a more compact spatial distribution characteristic compared to GWO, with shorter path node spacing and smoother steering angle. The convergence curve further reveals its dynamic performance advantage: MCEHBA rapidly approaches the optimal solution at the beginning of iterations (0–100 times), and the final adaptation value stabilises at 124.49, which is significantly better than the convergence trajectories of other algorithms.

#### 5.3.2. Analysis of Results for Map M2

As shown in Table 12, the path planning performance of MCEHBA in the complex environment M2 demonstrates an overall advantage. From the planning result data, the mean value of MCEHBA (208.67) is significantly lower than the other compared algorithms, and 43.6%, 40.7% and 28.4% lower than HBA (369.75), DOA (351.78), and GWO (291.35), respectively, which indicates that it has the highest optimisation efficiency, its best (175.07) and worst (217.07) values are equally better than all the algorithms, and it is especially noteworthy that MCEHBA’s worst (217.07) value is even equal to the best (217.07) value of DOA, this highlights the superior performance of MCEHBA in solving the path planning problem.

**Figure 10:**
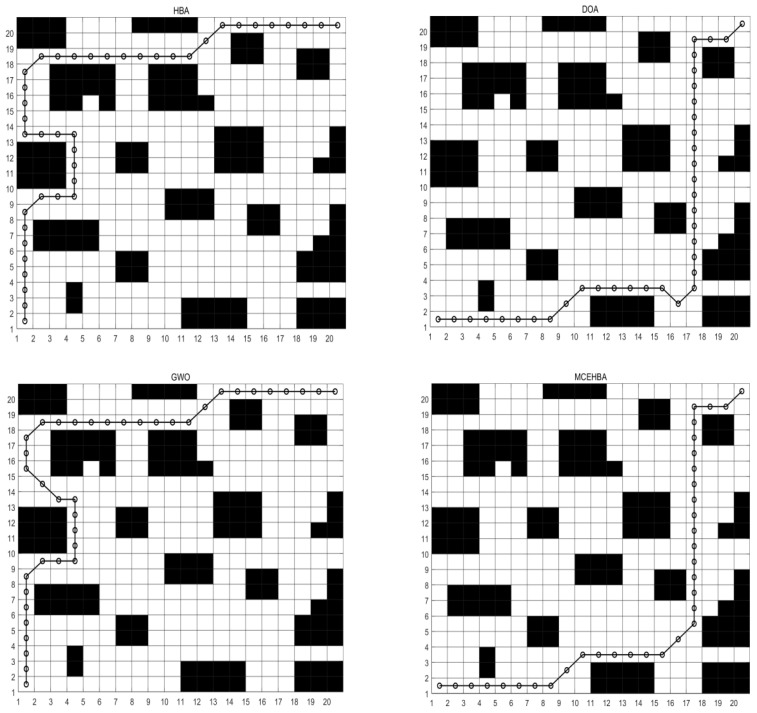
Route map of M2. (The black parts represent obstacles, and the black lines represent the motion paths planned by the robot).

**Figure 11:**
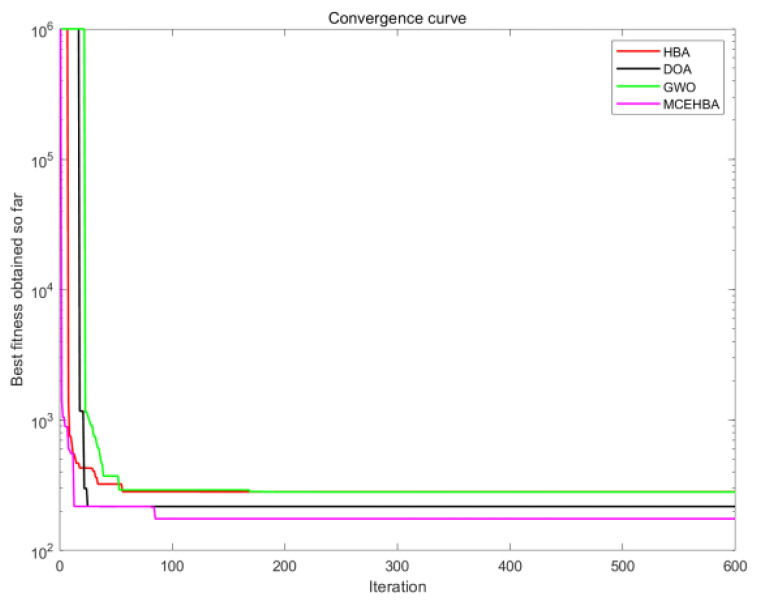
Convergence diagram of M2.

Combined with the analysis of Figure 10, paths planned with MCEHBA are more spatially efficient. Its path nodes are more densely distributed with fewer turning points and smoother angles, effectively reducing unnecessary energy consumption and time loss. Figure 11 further reveals the dynamic performance advantages of MCEHBA. During the initial iteration phase (0–100 iterations), the fitness value of MCEHBA rapidly declines, demonstrating its strong global search capability to quickly locate near-optimal solutions. In contrast, algorithms like GWO and HBA tend to prematurely converge to suboptimal solutions, leading to performance degradation.

#### 5.3.3. Analysis of Results for Map M3

**Figure 12:**
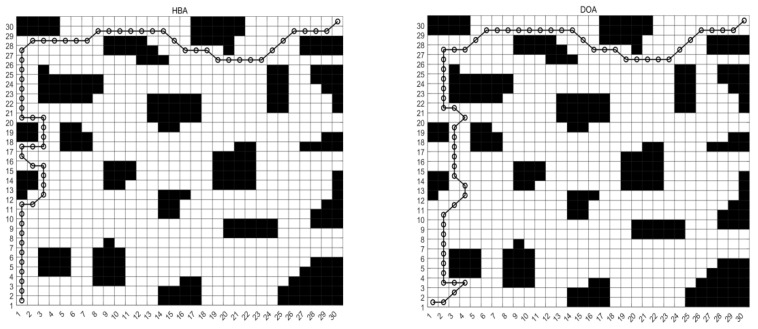

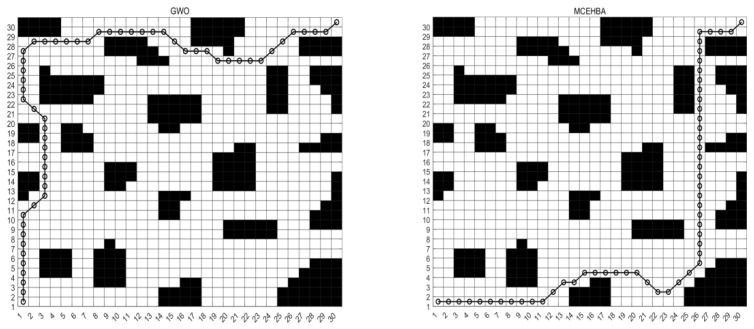
Route map of M3. (The black parts represent obstacles, and the black lines represent the motion paths planned by the robot).

**Figure 13:**
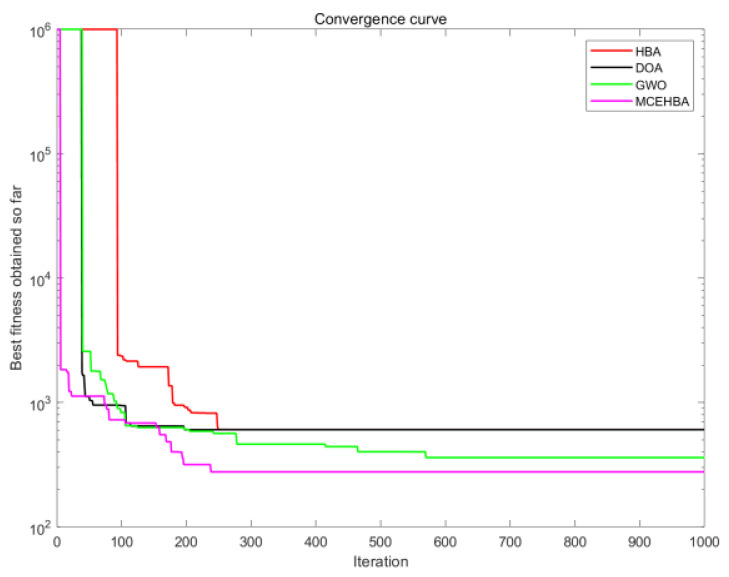
Convergence diagram of M3.

From Table 13, it can be analysed that MCEHBA shows a significant overall advantage in the path planning task, and its performance leads in many aspects compared to the other compared algorithms. In terms of mean value, MCEHBA slightly outperforms GWO (469.16) with a value of 465.00 and significantly outperforms HBA (748.23) and DOA (761.85), indicating its superiority in average planning efficiency. However, its standard deviation is as high as 17,319.76, which is larger than GWO’s 10,036.71, indicating some fluctuations in the stability of the algorithm. The optimal value of MCEHBA (276.73) is much lower than that of the other algorithms, highlighting its superior optimisation ability and proving that MCEHBA possesses a stronger local search capability with a more efficient convergence mechanism. Figure 12 and Figure 13 show that MCEHBA is able to plan a moving path with low cost and few redundant path points, has faster convergence speed, and is able to approach the optimal solution in fewer iterations, whereas DOA and HBA may converge slowly or fall into a local optimum due to algorithm design problems.

## 6. Discussion

### 6.1. Experimental Result

The ablation study results in Section 4.2 confirm the effectiveness of all improvement strategies adopted in MCEHBA. Variants 3, 4, and 5 consistently outperformed the control groups lacking their corresponding strategies, with significant performance gains. At a dimensionality of 30, Variants 3, 4, and 5 demonstrated superior performance on no fewer than 20 benchmark functions each. When dimensionality increased to 50, all three variants maintained advantages on approximately 20 functions. Notably, Variant 4 (incorporating differential mutation) surpassed the control group on 26 functions, validating the efficacy of the dispersed boundary handling strategy, the differential mutation strategy, and the centroid opposition-based learning strategy.

Regarding Variant 1, which implemented the nonlinear convergence factor strategy based on a sinusoidal function, while it exhibited no global optimisation advantage across the full set of 29 functions, experimental analysis revealed significant benefits: at 30 dimensions, it enhanced convergence efficiency for 57% of non-convex functions; at 50 dimensions, it delivered excellent performance on 50% of composite functions. Furthermore, Variant 2 (incorporating the good point set initialisation strategy) showed no overall performance improvement at 30 dimensions, yet still achieved outstanding results on 50% of hybrid functions and 50% of composite functions. These findings substantiate a fundamental principle of algorithm optimisation: no universal strategy exists; only methods adapted to specific problem characteristics are effective. This discovery offers a new direction for bio-inspired algorithm applications: strategy combinations can be freely selected based on the problem type being solved.

Comparative analysis demonstrates MCEHBA’s exceptional global search capability. As presented in Table 5, MCEHBA achieved optimal mean fitness values on 20 out of 29 test functions. Its superior comprehensive performance is further confirmed by the average rank (1.62) obtained from the Friedman test. As the Friedman test results were significant, a post-hoc Nemenyi test was conducted, revealing that MCEHBA significantly outperformed DOA, DBO, and WOA. For algorithms showing no significant difference from MCEHBA (HBA, GWO, and SO), the Friedman average ranks still indicate MCEHBA’s comparative advantage. It should be noted that MCEHBA failed to converge on function F24. Analysis suggests this may relate to the non-separable nature of variables introduced by function rotation, where the algorithm struggles to locate the global optimum—an issue requiring targeted improvement in future work. Nevertheless, MCEHBA exhibited robust convergence characteristics on the remaining 28 functions and maintained optimal performance across all hybrid functions.

### 6.2. Application to Engineering Problems

In this paper, by introducing the welded beam design, reducer design and the cantilever beam design problems, MCEHBA’s optimisation performance is systematically verified, and the experimental results show that MCEHBA has excellent results in solving engineering application problems. The welded beam design problem has 7 constraints, and MCEHBA gives the optimal solution among all algorithms, with a design cost of 1.69277; the reducer design problem has 11 constraints, and MCEHBA also gives the optimal solution with a volume of 2994.47, which fully demonstrates that MCEHBA shows excellent global search capability and constraint handling ability when solving engineering optimization problems with strong constraints. It further demonstrates the significance of the GCOBL and the differential evolution strategy, which are the two core improvement strategies that enable MCEHBA to exhibit significant performance advantages in engineering optimisation scenarios.

### 6.3. Experimental Analysis of Mobile Robot Path Planning

In the simulation experiment, in order to comprehensively test the path planning effect of MCEHBA in simple, medium and complex environments, this study designed three kinds of raster maps with increasing difficulty of path planning, and selected three typical group intelligence algorithms, namely, HBA, GWO and DOA, as the benchmark objects for comparison, which cover different optimization paradigms based on population collaboration, biologically-inspired search, and social behaviour simulation, with the characteristics of algorithmic diversity. Five independent experiments are performed for each scenario to quantify the stability of the algorithms through the mean, standard deviation, worst value and best value of the total path cost, while the total path cost is recorded to assess the energy optimality. The experimental results show that MCEHBA is able to find the optimal path with the lowest total cost; in particular, in complex environments, When the comparison algorithm is trapped by a locally optimal solution, MCEHBA effectively breaks through the convergence stagnation problem of traditional algorithms in dynamic obstacle avoidance by combining the mirror solution space generated by the centre of gravity reverse learning strategy through the DE/current-to-best/1 mutation mechanism to drive the directed search of the population to the neighbourhood of elite individuals.

## 7. Conclusions

For the needs of autonomous navigation of intelligent devices in the agricultural field, this paper introduces a good point set initialisation strategy and a decentralised boundary crossing processing strategy on the basis of the classical Honey Badger Algorithm (HBA), constructs a sinusoidal function-based nonlinear convergence factor, optimises the individual position updating phase by combining the differential variation strategy of the Differential Evolutionary Algorithm (DEA) with the centre of gravity back learning (GCOBL) strategy, and obtains an algorithm with improved performance. Algorithm MCEHBA, and verified it in various aspects, including the feasibility verification of the improved strategy, algorithm performance comparison experiments, convergence analysis, Friedman’s test, Nemenyi test, and engineering design problem experiments, and MCEHBA performs well in the above experiments, and finally MCEHBA is applied to simulation path planning experiments, and three path planning graphs with increasing difficulty are designed and compared with other three excellent swarm intelligence algorithms were compared. The results show that MCEHBA can find the solution that minimises the objective function stably and quickly, which proves the excellent performance of MCEHBA in solving optimisation problems, and in the future, MCEHBA can be applied to optimisation problems such as path planning or parameter optimisation in other scenarios.

However, MCEHBA still has performance flaws in specific scenarios. In the convergence analysis, it is found that MCEHBA cannot converge on the F24 function in the CEC2017 test function set, which may be due to the inseparability of the rotational variables in the F24 function, which makes it difficult to search for the optimal solution through the existing strategies, thus leading to the convergence stagnation phenomenon. As mentioned in the discussion, this issue will continue to be improved and optimised in the future. At the same time, in response to the problem of grid size affecting planning accuracy in the raster method (as described in Section 3.2), static grid configurations inevitably impose constraints on balancing precision and efficiency. To systematically address this challenge, our future work will prioritise the development and integration of adaptive mesh strategies. This approach operates through an iterative refinement mechanism: initial path exploration will occur on a coarse-grained grid to rapidly identify promising regions while minimising computational overhead. Subsequently, local mesh refinement will be triggered within these candidate areas for instance, by subdividing critical cells and their adjacent counterparts by a factor of two or more. This facilitates high-resolution, concentrated searches near potential optimal paths in subsequent phases. Consforuently, the precision of final trajectories is significantly enhanced without incurring the substantial costs associated with universally fine discretisation.

## Figures and Tables

**Table 1:** Initialisation Strategy Performance Evaluation Results.

		Average Nearest-Neighbour Distance	Distance Standard Deviation	Minimum Inter-Individual Distance	Spatial Coverage Ratio
**Dim = 3**	Good Point Set-based Population Initialization	**2.884**	**0.56188**	**2.2361**	1
	Random Initialization	2.4683	1.0468	0.43957	1
**Dim = 5**	Good Point Set-based Population Initialization	**7.0292**	**0.78513**	**5.4772**	**1**
	Random Initialization	5.8595	1.8657	2.3788	0.9375
**Dim = 10**	Good Point Set-based Population Initialization	**13.612**	**1.6305**	**11.314**	**0.09375**
	Random Initialization	13.085	2.1067	9.1448	0.09082

**Table 2:** Number of times an individual solution vector crosses the boundary.

Number of Runs	Average Number of Crossings	Maximum Number of Crossings
**1**	3.1960	222
**2**	2.8670	244
**3**	3.3050	205
**4**	3.49320	217
**5**	3.8010	260

**Table 3:** Results of ablation experiments when dim = 30.

	Mean	Std	Max	Min
HBA_Variant1	11	9	10	7
HBA_Variant2	13	18	10	12
HBA_Variant3	20	19	14	18
HBA_Variant4	25	18	21	23
HBA_Variant5	20	17	15	15

**Table 4:** Results of ablation experiments when dim = 50.

	Mean	Std	Max	Min
HBA_Variant1	7	15	8	8
HBA_Variant2	15	18	11	18
HBA_Variant3	19	15	18	13
HBA_Variant4	26	20	19	25
HBA_Variant5	20	17	17	14

**Table 5:** Performance Testing of MCEHBA.

	Mean	Std	Max	Min
F1~F3	2	1	2	2
F4~F10	2	1	3	1
F11~F20	10	8	9	8
F21~F30	6	5	6	5

**Table 6:** Friedman test results.

Algorithm	HBA	DOA	GWO	DBO	MCEHBA	SO	WOA
**Average rank**	2.9310	6.7586	3.2414	4.6897	1.6207	2.6552	6.1034

**Table 7:** Welding beam design issues test result.

Algorithms	x_1_	x_2_	x_3_	x_4_	Optima Quality
**DBO**	0.201	2.942	10.000	0.201	1.77317
**GWO**	0.204	3.265	9.034	0.206	1.69554
**SO**	0.205	3.242	9.038	0.206	1.69342
**DOA**	0.206	3.237	9.036	0.206	1.69315
**HBA**	0.206	3.221	9.079	0.206	1.69612
**MCEHBA**	0.206	3.235	9.037	0.206	1.69277

**Table 8:** Reducer design issues test result.

Algorithms	x_1_	x_2_	x_3_	x_4_	x_5_	x_6_	x_7_	Optima Quality
**DBO**	3.600	0.700	17.000	7.300	8.300	3.350	5.287	3.04671 × 10^3^
**GWO**	3.503	0.700	17.000	7.520	7.896	3.374	5.288	3.00843 × 10^3^
**SO**	3.500	0.700	17.000	7.300	7.715	3.350	5.287	2.99453 × 10^3^
**DOA**	3.500	0.700	17.000	7.300	7.717	3.350	5.287	2.99450 × 10^3^
**HBA**	3.500	0.700	17.000	7.300	7.715	3.350	5.287	2.99456 × 10^3^
**MCEHBA**	3.500	0.700	17.000	7.300	7.715	3.350	5.287	2.99447 × 10^3^
**WOA**	3.500	0.700	20.672	7.622	8.043	3.807	5.500	3.97481 × 10^3^

**Table 9:** Cantilever beam design issues test result.

Algorithms	x_1_	x_2_	x_3_	x_4_	x_5_	Optima Quality
**DBO**	5.99684	5.30211	4.50314	3.49623	2.17610	1.34000
**GWO**	5.98316	5.32172	4.48639	3.53607	2.14826	1.34008
**SO**	6.04859	5.29833	4.48870	3.50432	2.13451	1.34001
**DOA**	5.13085	4.37832	8.55756	6.79829	2.63024	1.71570
**HBA**	6.03167	5.32599	4.47873	3.48725	2.15043	1.33998
**MCEHBA**	6.01602	5.30917	4.49433	3.50147	2.15267	**1.33996**
**WOA**	6.20937	5.57908	4.87091	2.80830	2.68476	1.38231

**Table 10:** Map Information.

Map	Grid Specification	Start Position	End Position	Number of Obstacles	Obstacles Percent
**M1**	15 × 15	(1,1)	(15,15)	58	26%
**M2**	20 × 20	(1,1)	(20,20)	109	27%
**M3**	30 × 30	(1,1)	(30,30)	231	26%

**Table 11:** Planning results for each algorithm on M1.

Algorithm	Mean	Std	Best	Worst
**HBA**	183.33	1056.90	146.24	232.83
**DOA**	256.72	3996.19	168.24	317.90
**GWO**	158.34	358.93	145.66	189.07
**MCEHBA**	**140.95**	508.53	**124.49**	**165.66**

**Table 12:** Planning results for each algorithm on M2.

Algorithm	Mean	Std	Best	Worst
**HBA**	369.75	12,698.14	281.66	543.90
**DOA**	351.78	21,816.96	217.07	493.31
**GWO**	291.35	479.20	280.49	330.00
**MCEHBA**	**208.67**	**352.80**	**175.07**	**217.07**

**Table 13:** Planning results for each algorithm on M3.

Algorithm	Mean	Std	Best	Worst
**HBA**	748.23	42,414.43	605.56	1067.60
**DOA**	761.85	23,951.88	604.63	995.21
**GWO**	469.16	10,036.71	360.38	611.80
**MCEHBA**	465.00	17,319.76	276.73	619.01

**Figure A1:**
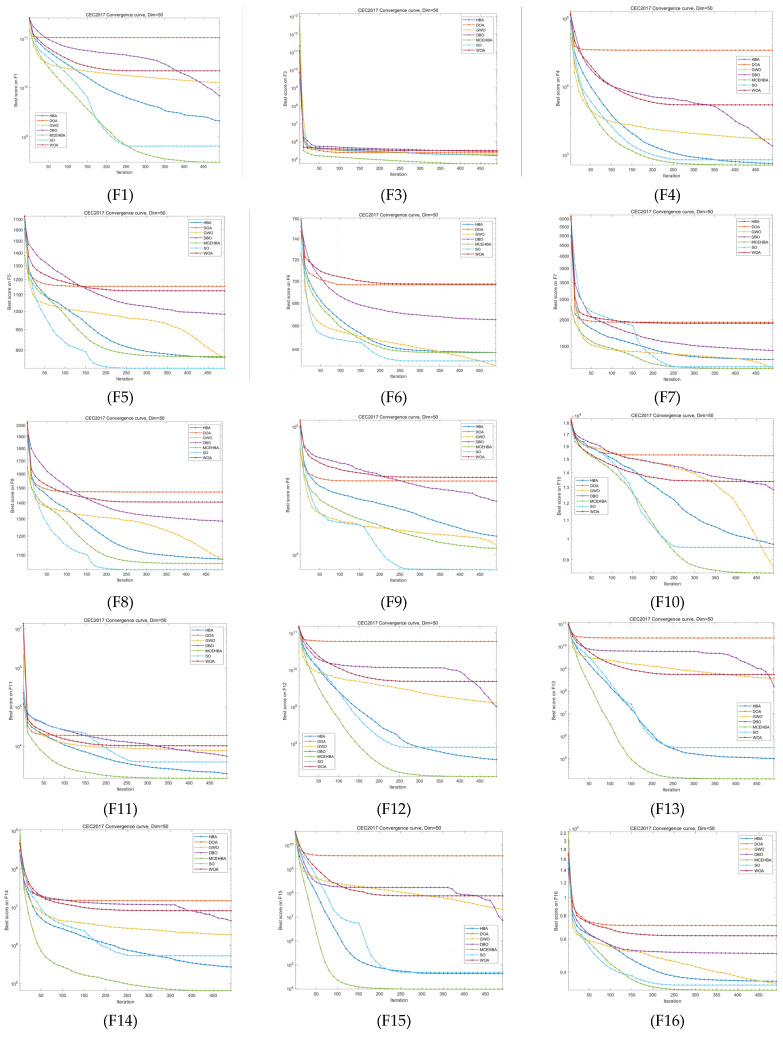

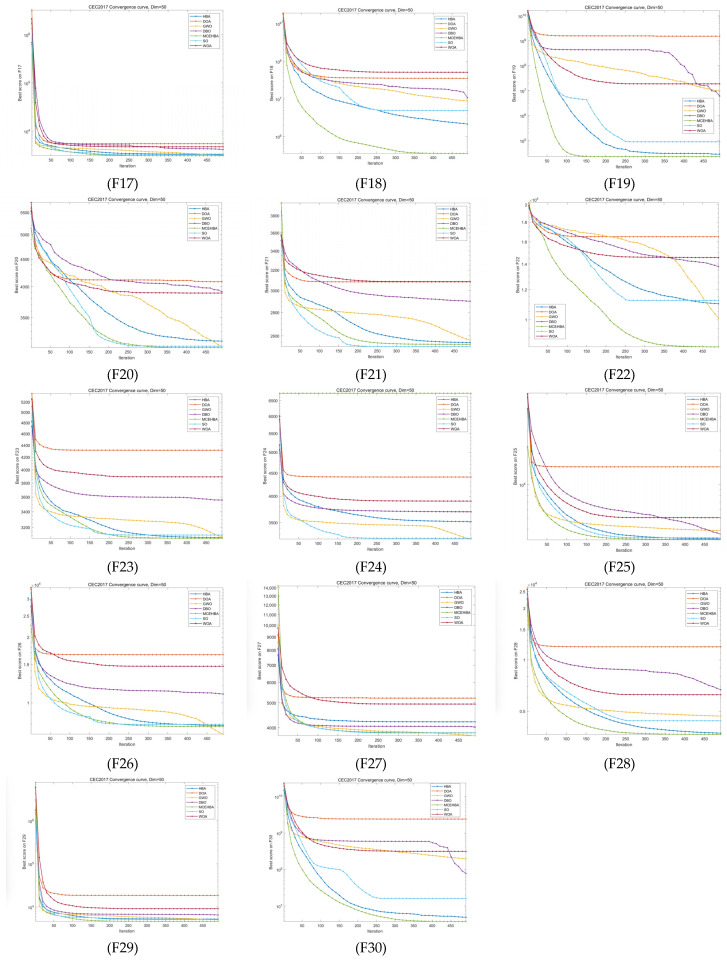
The complete convergence diagram.

## Data Availability

Data are contained within the article.

## Appendix A

**Table A1:** Dim = 30.

Function	Algorithm	Mean	Std	Max	Min
F1	HBA	1.3862 × 10^6^	4.8147 × 10^12^	1.1311 × 10^7^	4.9457 × 10^4^
	HBA Variant1	4.8878 × 10^7^	8.0296 × 10^15^	4.8894 × 10^8^	5.0715 × 10^6^
	HBA Variant2	**4.0339 × 10^7^**	3.3164 × 10^15^	3.2462 × 10^8^	6.1327 × 10^6^
	HBA Variant3	5.0328 × 10^7^	4.1998 × 10^15^	3.3773 × 10^8^	7.0629 × 10^6^
	HBA Variant4	9.1174 × 10^5^	1.3859 × 10^12^	5.8414 × 10^6^	3.8087 × 10^4^
	HBA Variant5	7.3168 × 10^5^	8.2264 × 10^11^	4.2273 × 10^6^	5.3006 × 10^4^
F3	HBA	3.7048 × 10^4^	9.0918 × 10^7^	5.2058 × 10^4^	2.0063 × 10^4^
	HBA Variant1	4.8771 × 10^4^	9.3261 × 10^7^	6.3413 × 10^4^	3.1881 × 10^4^
	HBA Variant2	5.0196 × 10^4^	7.7317 × 10^7^	6.8128 × 10^4^	3.0939 × 10^4^
	HBA Variant3	4.4646 × 10^4^	5.7147 × 10^7^	6.0493 × 10^4^	2.7099 × 10^4^
	HBA Variant4	6.5007 × 10^3^	5.4666 × 10^6^	1.1888 × 10^4^	2.4813 × 10^3^
	HBA Variant5	6.9880 × 10^3^	1.0537 × 10^7^	1.6645 × 10^4^	2.1950 × 10^3^
F4	HBA	5.2316 × 10^2^	1.3850 × 10^3^	6.2472 × 10^2^	4.6852 × 10^2^
	HBA Variant1	5.4563 × 10^2^	1.9361 × 10^3^	6.6362 × 10^2^	4.8365 × 10^2^
	HBA Variant2	**5.3965 × 10^2^**	1.5247 × 10^3^	6.1992 × 10^2^	4.7020 × 10^2^
	HBA Variant3	5.4881 × 10^2^	2.2237 × 10^3^	7.0534 × 10^2^	4.2635 × 10^2^
	HBA Variant4	5.2034 × 10^2^	1.2148 × 10^3^	6.0435 × 10^2^	4.0503 × 10^2^
	HBA Variant5	5.1885 × 10^2^	9.0008 × 10^2^	5.8219 × 10^2^	4.0139 × 10^2^
F5	HBA	6.2384 × 10^2^	4.9642 × 10^2^	6.8025 × 10^2^	5.7998 × 10^2^
	HBA Variant1	6.4277 × 10^2^	8.2536 × 10^2^	6.9012 × 10^2^	5.9075 × 10^2^
	HBA Variant2	6.4284 × 10^2^	1.0954 × 10^3^	7.3800 × 10^2^	6.0166 × 10^2^
	HBA Variant3	6.5350 × 10^2^	9.9217 × 10^2^	7.5263 × 10^2^	5.9910 × 10^2^
	HBA Variant4	6.3692 × 10^2^	1.2028 × 10^3^	7.1593 × 10^2^	5.7404 × 10^2^
	HBA Variant5	6.3508 × 10^2^	1.9502 × 10^3^	7.6182 × 10^2^	5.7465 × 10^2^
F6	HBA	6.2329 × 10^2^	1.0543 × 10^2^	6.4516 × 10^2^	6.0785 × 10^2^
	HBA Variant1	**6.2155 × 10^2^**	4.7340 × 10^1^	6.3697 × 10^2^	6.1053 × 10^2^
	HBA Variant2	**6.2129 × 10^2^**	5.7471 × 10^1^	6.3922 × 10^2^	6.1061 × 10^2^
	HBA Variant3	6.1946 × 10^2^	7.3767 × 10^1^	6.3982 × 10^2^	6.0672 × 10^2^
	HBA Variant4	6.1869 × 10^2^	7.0832 × 10^1^	6.4035 × 10^2^	6.0391 × 10^2^
	HBA Variant5	6.1705 × 10^2^	7.6460 × 10^1^	6.3763 × 10^2^	6.0541 × 10^2^
F7	HBA	9.4520 × 10^2^	6.4003 × 10^3^	1.2714 × 10^3^	8.4463 × 10^2^
	HBA Variant1	**9.4074 × 10^2^**	3.3671 × 10^3^	1.1298 × 10^3^	8.5869 × 10^2^
	HBA Variant2	9.5635 × 10^2^	5.7762 × 10^3^	1.2194 × 10^3^	8.4625 × 10^2^
	HBA Variant3	9.4375 × 10^2^	1.6664 × 10^3^	1.0176 × 10^3^	8.5138 × 10^2^
	HBA Variant4	9.1803 × 10^2^	2.6011 × 10^3^	1.0267 × 10^3^	8.3085 × 10^2^
	HBA Variant5	9.0054 × 10^2^	2.4875 × 10^3^	1.0136 × 10^3^	8.1588 × 10^2^
F8	HBA	9.1245 × 10^2^	5.4565 × 10^2^	9.5539 × 10^2^	8.6413 × 10^2^
	HBA Variant1	**9.1016 × 10^2^**	3.8984 × 10^2^	9.4795 × 10^2^	8.7267 × 10^2^
	HBA Variant2	9.1394 × 10^2^	5.7920 × 10^2^	9.8906 × 10^2^	8.7454 × 10^2^
	HBA Variant3	9.1793 × 10^2^	4.4043 × 10^2^	9.7127 × 10^2^	8.7245 × 10^2^
	HBA Variant4	9.1202 × 10^2^	8.8778 × 10^2^	9.8209 × 10^2^	8.5786 × 10^2^
	HBA Variant5	9.1194 × 10^2^	6.8054 × 10^2^	9.7719 × 10^2^	8.5808 × 10^2^
F9	HBA	2.9927 × 10^3^	6.4966 × 10^5^	4.5387 × 10^3^	1.5269 × 10^3^
	HBA Variant1	3.4181 × 10^3^	1.3978 × 10^6^	6.3487 × 10^3^	1.1017 × 10^3^
	HBA Variant2	3.7441 × 10^3^	1.3693 × 10^6^	6.0017 × 10^3^	1.9450 × 10^3^
	HBA Variant3	3.8789 × 10^3^	1.3828 × 10^6^	5.8075 × 10^3^	2.2791 × 10^3^
	HBA Variant4	3.3905 × 10^3^	1.8256 × 10^6^	6.8447 × 10^3^	1.2144 × 10^3^
	HBA Variant5	2.4121 × 10^3^	1.0466 × 10^6^	5.5315 × 10^3^	1.1435 × 10^3^
F10	HBA	5.9393 × 10^3^	9.5724 × 10^5^	8.3012 × 10^3^	3.7704 × 10^3^
	HBA Variant1	**5.8671 × 10^3^**	8.2237 × 10^5^	7.5143 × 10^3^	3.9979 × 10^3^
	HBA Variant2	7.3379 × 10^3^	1.4038 × 10^6^	9.8564 × 10^3^	4.9224 × 10^3^
	HBA Variant3	5.8188 × 10^3^	8.1045 × 10^5^	7.5033 × 10^3^	4.1155 × 10^3^
	HBA Variant4	4.7215 × 10^3^	5.3222 × 10^5^	7.1113 × 10^3^	3.4016 × 10^3^
	HBA Variant5	4.8939 × 10^3^	4.7606 × 10^5^	6.2447 × 10^3^	3.0554 × 10^3^
F11	HBA	1.3115 × 10^3^	5.7245 × 10^3^	1.4789 × 10^3^	1.1946 × 10^3^
	HBA Variant1	1.3585 × 10^3^	7.7381 × 10^3^	1.6103 × 10^3^	1.2321 × 10^3^
	HBA Variant2	**1.3467 × 10^3^**	5.6262 × 10^3^	1.5377 × 10^3^	1.2325 × 10^3^
	HBA Variant3	**1.2577 × 10^3^**	1.9689 × 10^3^	1.3783 × 10^3^	1.1653 × 10^3^
	HBA Variant4	1.2158 × 10^3^	1.6123 × 10^3^	1.2944 × 10^3^	1.1444 × 10^3^
	HBA Variant5	1.2134 × 10^3^	1.5446 × 10^3^	1.3017 × 10^3^	1.1377 × 10^3^
F12	HBA	1.2043 × 10^6^	1.5928 × 10^12^	5.4333 × 10^6^	1.7308 × 10^5^
	HBA Variant1	6.8348 × 10^6^	4.9101 × 10^14^	1.2371 × 10^8^	2.2405 × 10^5^
	HBA Variant2	**3.3645 × 10^6^**	6.3026 × 10^12^	1.0333 × 10^7^	2.5984 × 10^5^
	HBA Variant3	2.4005 × 10^6^	2.9158 × 10^12^	6.7546 × 10^6^	2.6643 × 10^5^
	HBA Variant4	4.6747 × 10^5^	1.7247 × 10^11^	1.5554 × 10^6^	1.0682 × 10^4^
	HBA Variant5	5.3736 × 10^5^	5.9009 × 10^11^	3.7682 × 10^6^	5.5324 × 10^4^
F13	HBA	6.4088 × 10^4^	4.3024 × 10^9^	3.1612 × 10^5^	7.1596 × 10^3^
	HBA Variant1	6.7227 × 10^4^	4.4099 × 10^9^	2.4106 × 10^5^	1.0873 × 10^4^
	HBA Variant2	2.1013 × 10^5^	6.8448 × 10^11^	4.5757 × 10^6^	4.7554 × 10^3^
	HBA Variant3	1.5114 × 10^4^	2.1075 × 10^8^	6.3275 × 10^4^	2.4979 × 10^3^
	HBA Variant4	7.4475 × 10^3^	4.6140 × 10^7^	2.6650 × 10^4^	1.7433 × 10^3^
	HBA Variant5	9.4799 × 10^3^	7.2872 × 10^7^	3.6726 × 10^4^	1.8155 × 10^3^
F14	HBA	1.1243 × 10^5^	9.5711 × 10^10^	1.6729 × 10^6^	2.5775 × 10^3^
	HBA Variant1	**3.0269 × 10^4^**	5.1048 × 10^8^	9.5841 × 10^4^	3.8666 × 10^3^
	HBA Variant2	6.7335 × 10^4^	4.5896 × 10^9^	2.5758 × 10^5^	2.2304 × 10^3^
	HBA Variant3	4.5414 × 10^4^	2.5430 × 10^9^	2.2691 × 10^5^	2.3393 × 10^3^
	HBA Variant4	1.9726 × 10^3^	1.5684 × 10^6^	8.5177 × 10^3^	1.5848 × 10^3^
	HBA Variant5	2.9346 × 10^3^	8.9711 × 10^6^	1.3585 × 10^4^	1.5480 × 10^3^
F15	HBA	2.5842 × 10^4^	5.7854 × 10^8^	9.4914 × 10^4^	2.1140 × 10^3^
	HBA Variant1	2.7514 × 10^4^	2.1072 × 10^9^	2.2660 × 10^5^	3.5501 × 10^3^
	HBA Variant2	**1.8958 × 10^4^**	5.0565 × 10^8^	1.0730 × 10^5^	2.0780 × 10^3^
	HBA Variant3	3.7794 × 10^3^	8.0703 × 10^6^	1.1690 × 10^4^	1.6300 × 10^3^
	HBA Variant4	5.8591 × 10^3^	5.7937 × 10^7^	4.0340 × 10^4^	1.7376 × 10^3^
	HBA Variant5	5.0200 × 10^3^	4.3765 × 10^7^	2.9498 × 10^4^	1.7804 × 10^3^
F16	HBA	2.6382 × 10^3^	6.8473 × 10^4^	3.1965 × 10^3^	2.1707 × 10^3^
	HBA Variant1	2.7042 × 10^3^	7.1696 × 10^4^	3.1817 × 10^3^	2.1106 × 10^3^
	HBA Variant2	2.8302 × 10^3^	1.9730 × 10^5^	3.6613 × 10^3^	1.8779 × 10^3^
	HBA Variant3	2.7613 × 10^3^	8.6274 × 10^4^	3.4019 × 10^3^	2.1135 × 10^3^
	HBA Variant4	2.5731 × 10^3^	1.0722 × 10^5^	3.3434 × 10^3^	1.8458 × 10^3^
	HBA Variant5	2.5246 × 10^3^	9.6061 × 10^4^	3.4188 × 10^3^	2.0343 × 10^3^
F17	HBA	2.3734 × 10^3^	5.1266 × 10^4^	2.8894 × 10^3^	1.8822 × 10^3^
	HBA Variant1	**2.2832 × 10^3^**	5.9338 × 10^4^	2.6967 × 10^3^	1.8164 × 10^3^
	HBA Variant2	2.3493 × 10^3^	3.0839 × 10^4^	2.7949 × 10^3^	2.0998 × 10^3^
	HBA Variant3	2.1143 × 10^3^	3.1636 × 10^4^	2.4514 × 10^3^	1.8072 × 10^3^
	HBA Variant4	2.0742 × 10^3^	2.9886 × 10^4^	2.4795 × 10^3^	1.7934 × 10^3^
	HBA Variant5	2.0522 × 10^3^	3.8103 × 10^4^	2.4773 × 10^3^	1.7818 × 10^3^
F18	HBA	4.8340 × 10^5^	1.7652 × 10^11^	1.9252 × 10^6^	4.3467 × 10^4^
	HBA Variant1	7.0416 × 10^5^	3.9114 × 10^11^	2.2578 × 10^6^	1.1225 × 10^5^
	HBA Variant2	**6.7983 × 10^5^**	5.6122 × 10^11^	2.8533 × 10^6^	1.2226 × 10^5^
	HBA Variant3	5.4733 × 10^5^	2.4949 × 10^11^	2.2294 × 10^6^	9.9536 × 10^4^
	HBA Variant4	8.1649 × 10^4^	9.7781 × 10^9^	5.5578 × 10^5^	2.4019 × 10^4^
	HBA Variant5	5.7601 × 10^4^	1.0501 × 10^9^	1.2279 × 10^5^	1.0667 × 10^4^
F19	HBA	9.2271 × 10^3^	7.0112 × 10^7^	3.6353 × 10^4^	2.0802 × 10^3^
	HBA Variant1	1.3638 × 10^4^	2.0499 × 10^8^	5.6795 × 10^4^	2.0454 × 10^3^
	HBA Variant2	**8.1209 × 10^3^**	4.1089 × 10^7^	2.8318 × 10^4^	2.1483 × 10^3^
	HBA Variant3	7.1488 × 10^3^	5.2206 × 10^7^	2.9141 × 10^4^	2.0006 × 10^3^
	HBA Variant4	7.0894 × 10^3^	1.8557 × 10^7^	1.9631 × 10^4^	2.3233 × 10^3^
	HBA Variant5	5.7822 × 10^3^	1.7429 × 10^7^	2.1258 × 10^4^	1.9565 × 10^3^
F20	HBA	2.5873 × 10^3^	4.0495 × 10^4^	2.9609 × 10^3^	2.1565 × 10^3^
	HBA Variant1	**2.5826 × 10^3^**	6.4201 × 10^4^	3.2215 × 10^3^	2.2328 × 10^3^
	HBA Variant2	2.6036 × 10^3^	6.8734 × 10^4^	3.2394 × 10^3^	2.2626 × 10^3^
	HBA Variant3	2.5301 × 10^3^	5.2664 × 10^4^	3.0441 × 10^3^	2.1717 × 10^3^
	HBA Variant4	2.4494 × 10^3^	2.6022 × 10^4^	2.7886 × 10^3^	2.1900 × 10^3^
	HBA Variant5	2.3716 × 10^3^	2.3915 × 10^4^	2.7345 × 10^3^	2.1297 × 10^3^
F21	HBA	2.4151 × 10^3^	8.9075 × 10^2^	2.4915 × 10^3^	2.3705 × 10^3^
	HBA Variant1	**2.4129 × 10^3^**	1.1507 × 10^3^	2.5017 × 10^3^	2.3514 × 10^3^
	HBA Variant2	2.4338 × 10^3^	1.0399 × 10^3^	2.5019 × 10^3^	2.3771 × 10^3^
	HBA Variant3	2.4171 × 10^3^	7.1525 × 10^2^	2.4723 × 10^3^	2.3654 × 10^3^
	HBA Variant4	2.4060 × 10^3^	1.9445 × 10^3^	2.4567 × 10^3^	2.2119 × 10^3^
	HBA Variant5	2.4001 × 10^3^	2.1691 × 10^3^	2.4604 × 10^3^	2.2049 × 10^3^
F22	HBA	4.3976 × 10^3^	7.0546 × 10^6^	9.6617 × 10^3^	2.3036 × 10^3^
	HBA Variant1	4.6452 × 10^3^	7.5198 × 10^6^	1.0017 × 10^4^	2.3234 × 10^3^
	HBA Variant2	5.3374 × 10^3^	6.3039 × 10^6^	9.1000 × 10^3^	2.3193 × 10^3^
	HBA Variant3	2.3491 × 10^3^	2.5846 × 10^3^	2.5795 × 10^3^	2.3175 × 10^3^
	HBA Variant4	2.5636 × 10^3^	9.0240 × 10^5^	6.2373 × 10^3^	2.3031 × 10^3^
	HBA Variant5	2.3100 × 10^3^	1.9024 × 10^1^	2.3224 × 10^3^	2.3040 × 10^3^
F23	HBA	2.8036 × 10^3^	2.7949 × 10^3^	2.9676 × 10^3^	2.7197 × 10^3^
	HBA Variant1	**2.7958 × 10^3^**	3.0289 × 10^3^	2.9880 × 10^3^	2.7296 × 10^3^
	HBA Variant2	2.8103 × 10^3^	1.9534 × 10^3^	2.9221 × 10^3^	2.7305 × 10^3^
	HBA Variant3	2.7762 × 10^3^	1.0173 × 10^3^	2.8377 × 10^3^	2.7135 × 10^3^
	HBA Variant4	2.7923 × 10^3^	2.3161 × 10^3^	2.8983 × 10^3^	2.7143 × 10^3^
	HBA Variant5	2.8092 × 10^3^	3.3510 × 10^3^	2.9459 × 10^3^	2.7040 × 10^3^
F24	HBA	3.1134 × 10^3^	4.6835 × 10^4^	3.6330 × 10^3^	2.9171 × 10^3^
	HBA Variant1	**3.0394 × 10^3^**	3.0111 × 10^4^	3.5665 × 10^3^	2.8937 × 10^3^
	HBA Variant2	4.6886 × 10^3^	8.5570 × 10^−25^	4.6886 × 10^3^	4.6886 × 10^3^
	HBA Variant3	4.6886 × 10^3^	8.5570 × 10^−25^	4.6886 × 10^3^	4.6886 × 10^3^
	HBA Variant4	4.6886 × 10^3^	8.5570 × 10^−25^	4.6886 × 10^3^	4.6886 × 10^3^
	HBA Variant5	4.6886 × 10^3^	8.5570 × 10^−25^	4.6886 × 10^3^	4.6886 × 10^3^
F25	HBA	2.9225 × 10^3^	4.1271 × 10^2^	2.9615 × 10^3^	2.8866 × 10^3^
	HBA Variant1	2.9364 × 10^3^	6.6324 × 10^2^	3.0001 × 10^3^	2.9014 × 10^3^
	HBA Variant2	**2.9328 × 10^3^**	8.2271 × 10^2^	2.9966 × 10^3^	2.8914 × 10^3^
	HBA Variant3	2.9496 × 10^3^	7.8584 × 10^2^	2.9949 × 10^3^	2.9002 × 10^3^
	HBA Variant4	2.9063 × 10^3^	2.6469 × 10^2^	2.9519 × 10^3^	2.8843 × 10^3^
	HBA Variant5	2.9152 × 10^3^	6.1532 × 10^2^	2.9763 × 10^3^	2.8862 × 10^3^
F26	HBA	4.6724 × 10^3^	9.7823 × 10^5^	6.2253 × 10^3^	2.8146 × 10^3^
	HBA Variant1	4.8338 × 10^3^	7.4366 × 10^5^	6.3587 × 10^3^	2.9037 × 10^3^
	HBA Variant2	2.4117 × 10^4^	1.2322 × 10^−22^	2.4117 × 10^4^	2.4117 × 10^4^
	HBA Variant3	4.7731 × 10^3^	1.2321 × 10^6^	7.0976 × 10^3^	3.0021 × 10^3^
	HBA Variant4	4.6017 × 10^3^	2.0687 × 10^6^	7.0404 × 10^3^	2.8131 × 10^3^
	HBA Variant5	3.9026 × 10^3^	1.3160 × 10^6^	6.0779 × 10^3^	2.8174 × 10^3^
F27	HBA	3.3714 × 10^3^	2.6669 × 10^4^	3.8894 × 10^3^	3.2263 × 10^3^
	HBA Variant1	3.4460 × 10^3^	5.9532 × 10^4^	4.4299 × 10^3^	3.2246 × 10^3^
	HBA Variant2	**3.2997 × 10^3^**	1.2106 × 10^4^	3.5876 × 10^3^	3.1987 × 10^3^
	HBA Variant3	3.2843 × 10^3^	1.0950 × 10^3^	3.3657 × 10^3^	3.2212 × 10^3^
	HBA Variant4	3.2792 × 10^3^	8.2332 × 10^2^	3.3409 × 10^3^	3.2285 × 10^3^
	HBA Variant5	3.2785 × 10^3^	3.1194 × 10^3^	3.5381 × 10^3^	3.2105 × 10^3^
F28	HBA	3.2870 × 10^3^	1.6450 × 10^3^	3.4106 × 10^3^	3.2216 × 10^3^
	HBA Variant1	3.3152 × 10^3^	1.5978 × 10^3^	3.4204 × 10^3^	3.2261 × 10^3^
	HBA Variant2	**3.3107 × 10^3^**	1.0332 × 10^3^	3.3848 × 10^3^	3.2335 × 10^3^
	HBA Variant3	3.3304 × 10^3^	1.9616 × 10^3^	3.4000 × 10^3^	3.2391 × 10^3^
	HBA Variant4	3.2792 × 10^3^	1.5306 × 10^3^	3.4003 × 10^3^	3.2088 × 10^3^
	HBA Variant5	3.2645 × 10^3^	1.1055 × 10^3^	3.3257 × 10^3^	3.2088 × 10^3^
F29	HBA	4.3643 × 10^3^	1.7400 × 10^6^	1.0994 × 10^4^	3.5567 × 10^3^
	HBA Variant1	**4.1885 × 10^3^**	1.5076 × 10^5^	5.2167 × 10^3^	3.6182 × 10^3^
	HBA Variant2	**4.0393 × 10^3^**	1.4524 × 10^5^	4.9735 × 10^3^	3.5628 × 10^3^
	HBA Variant3	3.9796 × 10^3^	7.4547 × 10^4^	4.7002 × 10^3^	3.4926 × 10^3^
	HBA Variant4	3.7984 × 10^3^	4.6123 × 10^4^	4.2896 × 10^3^	3.4504 × 10^3^
	HBA Variant5	3.8086 × 10^3^	3.5469 × 10^4^	4.3234 × 10^3^	3.4742 × 10^3^
F30	HBA	5.4288 × 10^4^	1.2039 × 10^10^	6.2052 × 10^5^	9.9590 × 10^3^
	HBA Variant1	1.4352 × 10^5^	8.2639 × 10^10^	1.5853 × 10^6^	1.7359 × 10^4^
	HBA Variant2	**4.8320 × 10^4^**	1.4924 × 10^9^	1.7983 × 10^5^	9.6910 × 10^3^
	HBA Variant3	8.4822 × 10^4^	5.4871 × 10^9^	2.7330 × 10^5^	9.7172 × 10^3^
	HBA Variant4	2.0187 × 10^4^	9.6273 × 10^7^	5.3792 × 10^4^	8.3677 × 10^3^
	HBA Variant5	1.6601 × 10^4^	3.9915 × 10^7^	3.2941 × 10^4^	7.9474 × 10^3^

**Table A2:** Dim = 50.

Function	Algorithm	Mean	Std	Max	Min
F1	HBA	1.5420 × 10^9^	2.9319 × 10^18^	7.9273 × 10^9^	9.2268 × 10^7^
	HBA Variant1	4.3994 × 10^9^	7.6230 × 10^18^	1.5690 × 10^10^	1.0936 × 10^9^
	HBA Variant2	4.0562 × 10^9^	5.7323 × 10^18^	9.8912 × 10^9^	8.4821 × 10^8^
	HBA Variant3	5.1972 × 10^9^	6.9166 × 10^18^	1.1306 × 10^10^	1.1365 × 10^9^
	HBA Variant4	4.4176 × 10^8^	1.3714 × 10^17^	1.4332 × 10^9^	5.2124 × 10^7^
	HBA Variant5	3.0458 × 10^8^	1.5907 × 10^17^	1.8927 × 10^9^	3.9061 × 10^7^
F3	HBA	1.4753 × 10^5^	7.0925 × 10^8^	2.2454 × 10^5^	1.0379 × 10^5^
	HBA Variant1	1.6343 × 10^5^	8.3193 × 10^8^	2.3151 × 10^5^	1.1614 × 10^5^
	HBA Variant2	1.5175 × 10^5^	9.5785 × 10^8^	2.4312 × 10^5^	8.8770 × 10^4^
	HBA Variant3	1.4264 × 10^5^	5.2094 × 10^8^	1.8500 × 10^5^	9.4709 × 10^4^
	HBA Variant4	5.9437 × 10^4^	1.7746 × 10^8^	8.4280 × 10^4^	3.0618 × 10^4^
	HBA Variant5	5.5989 × 10^4^	1.0850 × 10^8^	7.2889 × 10^4^	3.5091 × 10^4^
F4	HBA	7.7198 × 10^2^	2.0837 × 10^4^	1.3419 × 10^3^	6.0247 × 10^2^
	HBA Variant1	1.0104 × 10^3^	1.3296 × 10^5^	2.2274 × 10^3^	7.1127 × 10^2^
	HBA Variant2	9.8891 × 10^2^	8.3409 × 10^4^	2.0985 × 10^3^	6.9623 × 10^2^
	HBA Variant3	1.2230 × 10^3^	2.6035 × 10^5^	3.0537 × 10^3^	7.5173 × 10^2^
	HBA Variant4	7.3932 × 10^2^	8.9809 × 10^3^	9.7770 × 10^2^	5.4338 × 10^2^
	HBA Variant5	7.5184 × 10^2^	1.1592 × 10^4^	1.0542 × 10^3^	5.4453 × 10^2^
F5	HBA	7.6510 × 10^2^	2.0036 × 10^3^	8.5816 × 10^2^	6.6816 × 10^2^
	HBA Variant1	8.2566 × 10^2^	1.7193 × 10^3^	8.8534 × 10^2^	7.0449 × 10^2^
	HBA Variant2	8.2287 × 10^2^	9.0853 × 10^2^	9.0802 × 10^2^	7.6766 × 10^2^
	HBA Variant3	8.0607 × 10^2^	1.8046 × 10^3^	9.0061 × 10^2^	7.0756 × 10^2^
	HBA Variant4	7.7148 × 10^2^	9.6918 × 10^2^	8.2090 × 10^2^	7.1495 × 10^2^
	HBA Variant5	7.7220 × 10^2^	2.1177 × 10^3^	8.4962 × 10^2^	6.6483 × 10^2^
F6	HBA	6.3560 × 10^2^	7.7635 × 10^1^	6.5176 × 10^2^	6.2020 × 10^2^
	HBA Variant1	6.3521 × 10^2^	7.4970 × 10^1^	6.5313 × 10^2^	6.1879 × 10^2^
	HBA Variant2	6.3863 × 10^2^	6.7670 × 10^1^	6.5881 × 10^2^	6.1851 × 10^2^
	HBA Variant3	6.4141 × 10^2^	1.3547 × 10^2^	6.6539 × 10^2^	6.2207 × 10^2^
	HBA Variant4	6.3937 × 10^2^	2.0099 × 10^2^	6.6938 × 10^2^	6.1710 × 10^2^
	HBA Variant5	6.3457 × 10^2^	6.8766 × 10^1^	6.5506 × 10^2^	6.2216 × 10^2^
F7	HBA	1.2864 × 10^3^	1.3353 × 10^4^	1.4854 × 10^3^	1.0840 × 10^3^
	HBA Variant1	1.3178 × 10^3^	1.3195 × 10^4^	1.4863 × 10^3^	1.0658 × 10^3^
	HBA Variant2	1.3133 × 10^3^	1.1777 × 10^4^	1.5370 × 10^3^	1.1239 × 10^3^
	HBA Variant3	1.2948 × 10^3^	1.4598 × 10^4^	1.6446 × 10^3^	1.0943 × 10^3^
	HBA Variant4	1.2080 × 10^3^	6.5671 × 10^3^	1.4140 × 10^3^	1.0325 × 10^3^
	HBA Variant5	1.1949 × 10^3^	8.0008 × 10^3^	1.3444 × 10^3^	1.0191 × 10^3^
F8	HBA	1.0802 × 10^3^	1.7468 × 10^3^	1.1971 × 10^3^	1.0087 × 10^3^
	HBA Variant1	1.1228 × 10^3^	2.0112 × 10^3^	1.2130 × 10^3^	1.0288 × 10^3^
	HBA Variant2	1.1278 × 10^3^	2.7601 × 10^3^	1.2709 × 10^3^	1.0460 × 10^3^
	HBA Variant3	1.1109 × 10^3^	1.8336 × 10^3^	1.2031 × 10^3^	1.0142 × 10^3^
	HBA Variant4	1.0775 × 10^3^	2.5188 × 10^3^	1.2244 × 10^3^	9.7147 × 10^2^
	HBA Variant5	1.0529 × 10^3^	2.1677 × 10^3^	1.1424 × 10^3^	9.2712 × 10^2^
F9	HBA	1.4373 × 10^4^	3.8445 × 10^7^	3.7539 × 10^4^	7.0875 × 10^3^
	HBA Variant1	1.6236 × 10^4^	2.6071 × 10^7^	2.5698 × 10^4^	5.9126 × 10^3^
	HBA Variant2	1.6445 × 10^4^	4.1049 × 10^7^	3.1185 × 10^4^	4.8276 × 10^3^
	HBA Variant3	1.6441 × 10^4^	2.0685 × 10^7^	2.4615 × 10^4^	7.3867 × 10^3^
	HBA Variant4	1.1794 × 10^4^	3.3485 × 10^7^	2.9591 × 10^4^	4.2652 × 10^3^
	HBA Variant5	1.1379 × 10^4^	2.0399 × 10^7^	2.2884 × 10^4^	4.7137 × 10^3^
F10	HBA	9.5957 × 10^3^	4.2257 × 10^6^	1.5352 × 10^4^	6.7203 × 10^3^
	HBA Variant1	1.0738 × 10^4^	3.6324 × 10^6^	1.4632 × 10^4^	8.1139 × 10^3^
	HBA Variant2	1.0524 × 10^4^	2.8773 × 10^6^	1.4944 × 10^4^	8.0319 × 10^3^
	HBA Variant3	1.0225 × 10^4^	2.0784 × 10^6^	1.3294 × 10^4^	7.7728 × 10^3^
	HBA Variant4	8.5074 × 10^3^	1.2849 × 10^6^	1.0628 × 10^4^	6.1257 × 10^3^
	HBA Variant5	8.1310 × 10^3^	1.3567 × 10^6^	1.0104 × 10^4^	6.2622 × 10^3^
F11	HBA	1.6647 × 10^3^	3.5324 × 10^4^	2.1881 × 10^3^	1.4236 × 10^3^
	HBA Variant1	2.3969 × 10^3^	6.3243 × 10^5^	4.5283 × 10^3^	1.5908 × 10^3^
	HBA Variant2	2.6247 × 10^3^	8.3169 × 10^5^	4.9510 × 10^3^	1.6606 × 10^3^
	HBA Variant3	2.6781 × 10^3^	9.8280 × 10^5^	4.6356 × 10^3^	1.5248 × 10^3^
	HBA Variant4	1.4810 × 10^3^	1.3372 × 10^4^	1.8681 × 10^3^	1.3237 × 10^3^
	HBA Variant5	1.4753 × 10^3^	1.0159 × 10^4^	1.6648 × 10^3^	1.3264 × 10^3^
F12	HBA	2.7615 × 10^7^	6.0681 × 10^14^	1.3201 × 10^8^	4.4241 × 10^6^
	HBA Variant1	9.1168 × 10^7^	8.8000 × 10^15^	5.1703 × 10^8^	2.0054 × 10^7^
	HBA Variant2	8.2548 × 10^7^	2.1362 × 10^15^	2.0503 × 10^8^	1.8564 × 10^7^
	HBA Variant3	5.9247 × 10^7^	2.2496 × 10^15^	2.6457 × 10^8^	1.2663 × 10^7^
	HBA Variant4	1.5537 × 10^7^	6.9737 × 10^13^	3.6939 × 10^7^	2.8920 × 10^6^
	HBA Variant5	1.4543 × 10^7^	2.6946 × 10^14^	8.8085 × 10^7^	2.4369 × 10^6^
F13	HBA	8.4991 × 10^4^	5.3884 × 10^9^	3.7841 × 10^5^	9.8553 × 10^3^
	HBA Variant1	2.1660 × 10^5^	4.3302 × 10^10^	1.1016 × 10^6^	4.7312 × 10^4^
	HBA Variant2	4.0434 × 10^5^	6.4108 × 10^11^	4.2599 × 10^6^	2.9266 × 10^4^
	HBA Variant3	6.0195 × 10^4^	3.1417 × 10^9^	3.0138 × 10^5^	1.4066 × 10^4^
	HBA Variant4	1.6954 × 10^4^	2.9294 × 10^8^	9.6964 × 10^4^	4.9999 × 10^3^
	HBA Variant5	1.3275 × 10^4^	6.3661 × 10^7^	2.8982 × 10^4^	4.4306 × 10^3^
F14	HBA	1.1906 × 10^6^	2.1680 × 10^13^	2.5754 × 10^7^	1.7225 × 10^4^
	HBA Variant1	1.3705 × 10^6^	1.0488 × 10^13^	1.5497 × 10^7^	3.2540 × 10^4^
	HBA Variant2	6.8941 × 10^5^	7.1502 × 10^11^	4.2049 × 10^6^	2.9088 × 10^4^
	HBA Variant3	5.3568 × 10^5^	4.4727 × 10^11^	3.0549 × 10^6^	3.6012 × 10^4^
	HBA Variant4	5.6948 × 10^4^	3.0634 × 10^9^	2.4267 × 10^5^	2.1121 × 10^3^
	HBA Variant5	6.0714 × 10^4^	5.2733 × 10^9^	3.1058 × 10^5^	4.8267 × 10^3^
F15	HBA	3.2531 × 10^4^	3.5808 × 10^8^	7.5185 × 10^4^	4.2814 × 10^3^
	HBA Variant1	6.0829 × 10^4^	2.2671 × 10^9^	2.0867 × 10^5^	1.6457 × 10^4^
	HBA Variant2	5.1395 × 10^4^	9.5142 × 10^8^	1.1345 × 10^5^	1.3603 × 10^4^
	HBA Variant3	1.2195 × 10^4^	4.7574 × 10^7^	2.3958 × 10^4^	3.0609 × 10^3^
	HBA Variant4	1.2893 × 10^4^	5.5510 × 10^7^	3.3343 × 10^4^	2.2830 × 10^3^
	HBA Variant5	1.2561 × 10^4^	3.6241 × 10^7^	2.0081 × 10^4^	2.8071 × 10^3^
F16	HBA	3.5332 × 10^3^	2.8950 × 10^5^	4.5469 × 10^3^	2.3564 × 10^3^
	HBA Variant1	3.7011 × 10^3^	2.3320 × 10^5^	4.6593 × 10^3^	2.9155 × 10^3^
	HBA Variant2	3.7689 × 10^3^	1.7089 × 10^5^	4.6377 × 10^3^	2.9405 × 10^3^
	HBA Variant3	3.4917 × 10^3^	2.4914 × 10^5^	4.7043 × 10^3^	2.4389 × 10^3^
	HBA Variant4	3.2869 × 10^3^	1.3733 × 10^5^	3.9777 × 10^3^	2.6596 × 10^3^
	HBA Variant5	3.4083 × 10^3^	2.0681 × 10^5^	4.4871 × 10^3^	2.6545 × 10^3^
F17	HBA	3.3505 × 10^3^	1.6089 × 10^5^	4.2245 × 10^3^	2.7226 × 10^3^
	HBA Variant1	3.3817 × 10^3^	1.1554 × 10^5^	4.0499 × 10^3^	2.6649 × 10^3^
	HBA Variant2	3.3873 × 10^3^	1.7320 × 10^5^	4.1500 × 10^3^	2.6241 × 10^3^
	HBA Variant3	3.1655 × 10^3^	1.5801 × 10^5^	3.9809 × 10^3^	2.5251 × 10^3^
	HBA Variant4	3.0138 × 10^3^	9.8325 × 10^4^	3.6547 × 10^3^	2.3185 × 10^3^
	HBA Variant5	2.9825 × 10^3^	1.0718 × 10^5^	3.5434 × 10^3^	2.1352 × 10^3^
F18	HBA	2.3957 × 10^6^	3.2514 × 10^12^	7.5405 × 10^6^	1.4852 × 10^5^
	HBA Variant1	1.9991 × 10^6^	3.4318 × 10^12^	7.8341 × 10^6^	2.9004 × 10^5^
	HBA Variant2	2.9983 × 10^6^	4.5396 × 10^12^	9.2974 × 10^6^	2.5223 × 10^5^
	HBA Variant3	4.6479 × 10^6^	1.4414 × 10^13^	1.3091 × 10^7^	4.3063 × 10^5^
	HBA Variant4	4.7599 × 10^5^	2.4964 × 10^11^	2.5893 × 10^6^	5.0458 × 10^4^
	HBA Variant5	3.4289 × 10^5^	1.2778 × 10^11^	1.6395 × 10^6^	8.5556 × 10^4^
F19	HBA	2.0517 × 10^4^	1.7408 × 10^8^	4.4102 × 10^4^	2.3485 × 10^3^
	HBA Variant1	4.0120 × 10^4^	2.4844 × 10^9^	2.9001 × 10^5^	4.2240 × 10^3^
	HBA Variant2	2.7690 × 10^4^	2.6446 × 10^8^	6.9020 × 10^4^	5.7685 × 10^3^
	HBA Variant3	2.6046 × 10^4^	1.0823 × 10^8^	4.2168 × 10^4^	9.6255 × 10^3^
	HBA Variant4	2.0617 × 10^4^	1.0728 × 10^8^	4.2961 × 10^4^	5.6065 × 10^3^
	HBA Variant5	2.1263 × 10^4^	9.6453 × 10^7^	3.8900 × 10^4^	5.2193 × 10^3^
F20	HBA	3.1946 × 10^3^	1.6368 × 10^5^	4.1407 × 10^3^	2.5176 × 10^3^
	HBA Variant1	3.2834 × 10^3^	1.3258 × 10^5^	4.4361 × 10^3^	2.5626 × 10^3^
	HBA Variant2	3.4128 × 10^3^	1.2776 × 10^5^	4.0172 × 10^3^	2.6198 × 10^3^
	HBA Variant3	3.3963 × 10^3^	9.0278 × 10^4^	3.9169 × 10^3^	2.8710 × 10^3^
	HBA Variant4	3.2345 × 10^3^	8.9664 × 10^4^	3.7715 × 10^3^	2.7102 × 10^3^
	HBA Variant5	3.1219 × 10^3^	1.2596 × 10^5^	3.9370 × 10^3^	2.3455 × 10^3^
F21	HBA	2.5611 × 10^3^	2.8997 × 10^3^	2.6928 × 10^3^	2.4711 × 10^3^
	HBA Variant1	2.5820 × 10^3^	2.6362 × 10^3^	2.7207 × 10^3^	2.4582 × 10^3^
	HBA Variant2	2.5963 × 10^3^	2.6197 × 10^3^	2.7350 × 10^3^	2.5133 × 10^3^
	HBA Variant3	2.5724 × 10^3^	2.1062 × 10^3^	2.6653 × 10^3^	2.5027 × 10^3^
	HBA Variant4	2.5306 × 10^3^	1.9981 × 10^3^	2.6323 × 10^3^	2.4547 × 10^3^
	HBA Variant5	2.5418 × 10^3^	1.5864 × 10^3^	2.6752 × 10^3^	2.4739 × 10^3^
F22	HBA	1.1105 × 10^4^	4.4696 × 10^6^	1.4451 × 10^4^	3.9381 × 10^3^
	HBA Variant1	1.2229 × 10^4^	2.2453 × 10^6^	1.5538 × 10^4^	8.7929 × 10^3^
	HBA Variant2	1.2140 × 10^4^	5.5609 × 10^6^	1.5778 × 10^4^	2.7543 × 10^3^
	HBA Variant3	9.0497 × 10^3^	1.5793 × 10^7^	1.4149 × 10^4^	2.5039 × 10^3^
	HBA Variant4	8.6397 × 10^3^	1.2757 × 10^7^	1.2323 × 10^4^	2.3966 × 10^3^
	HBA Variant5	8.8657 × 10^3^	1.0709 × 10^7^	1.3459 × 10^4^	2.3911 × 10^3^
F23	HBA	3.0873 × 10^3^	8.9441 × 10^3^	3.3933 × 10^3^	2.9124 × 10^3^
	HBA Variant1	3.0757 × 10^3^	3.7157 × 10^3^	3.2409 × 10^3^	2.9663 × 10^3^
	HBA Variant2	3.0787 × 10^3^	4.8742 × 10^3^	3.2467 × 10^3^	2.9272 × 10^3^
	HBA Variant3	3.0714 × 10^3^	4.2311 × 10^3^	3.2085 × 10^3^	2.9328 × 10^3^
	HBA Variant4	3.0757 × 10^3^	4.6087 × 10^3^	3.2279 × 10^3^	2.9790 × 10^3^
	HBA Variant5	3.0728 × 10^3^	7.2032 × 10^3^	3.2729 × 10^3^	2.9227 × 10^3^
F24	HBA	3.5490 × 10^3^	2.1112 × 10^5^	4.5566 × 10^3^	3.1240 × 10^3^
	HBA Variant1	3.4566 × 10^3^	1.5542 × 10^5^	4.5077 × 10^3^	3.1018 × 10^3^
	HBA Variant2	6.7454 × 10^3^	8.5570 × 10^−25^	6.7454 × 10^3^	6.7454 × 10^3^
	HBA Variant3	6.7454 × 10^3^	8.5570 × 10^−25^	6.7454 × 10^3^	6.7454 × 10^3^
	HBA Variant4	6.7454 × 10^3^	8.5570 × 10^−25^	6.7454 × 10^3^	6.7454 × 10^3^
	HBA Variant5	6.7454 × 10^3^	8.5570 × 10^−25^	6.7454 × 10^3^	6.7454 × 10^3^
F25	HBA	3.2832 × 10^3^	1.0837 × 10^4^	3.5738 × 10^3^	3.1453 × 10^3^
	HBA Variant1	3.4689 × 10^3^	4.0923 × 10^4^	3.9423 × 10^3^	3.1541 × 10^3^
	HBA Variant2	3.4378 × 10^3^	3.6545 × 10^4^	3.9504 × 10^3^	3.2091 × 10^3^
	HBA Variant3	3.6478 × 10^3^	1.0866 × 10^5^	4.9441 × 10^3^	3.3252 × 10^3^
	HBA Variant4	3.2442 × 10^3^	5.2472 × 10^3^	3.4784 × 10^3^	3.1244 × 10^3^
	HBA Variant5	3.2287 × 10^3^	4.5178 × 10^3^	3.3893 × 10^3^	3.1160 × 10^3^
F26	HBA	7.9672 × 10^3^	2.4340 × 10^6^	1.1381 × 10^4^	5.7900 × 10^3^
	HBA Variant1	7.4303 × 10^3^	2.3268 × 10^6^	1.0814 × 10^4^	4.5991 × 10^3^
	HBA Variant2	7.4800 × 10^3^	5.8024 × 10^6^	1.2326 × 10^4^	4.4648 × 10^3^
	HBA Variant3	8.0229 × 10^3^	4.6671 × 10^6^	1.1135 × 10^4^	4.4430 × 10^3^
	HBA Variant4	6.8605 × 10^3^	6.9379 × 10^6^	1.2266 × 10^4^	3.8720 × 10^3^
	HBA Variant5	7.7758 × 10^3^	6.0474 × 10^6^	1.1017 × 10^4^	4.0915 × 10^3^
F27	HBA	4.3209 × 10^3^	2.9043 × 10^5^	5.5419 × 10^3^	3.6089 × 10^3^
	HBA Variant1	4.2962 × 10^3^	3.8208 × 10^5^	5.6456 × 10^3^	3.4706 × 10^3^
	HBA Variant2	4.0895 × 10^3^	2.8326 × 10^5^	5.4600 × 10^3^	3.4020 × 10^3^
	HBA Variant3	3.8483 × 10^3^	1.2146 × 10^4^	4.0791 × 10^3^	3.6599 × 10^3^
	HBA Variant4	3.8045 × 10^3^	2.2544 × 10^4^	4.2022 × 10^3^	3.5012 × 10^3^
	HBA Variant5	3.7640 × 10^3^	1.8584 × 10^4^	3.9939 × 10^3^	3.4823 × 10^3^
F28	HBA	3.7327 × 10^3^	3.4854 × 10^4^	4.1442 × 10^3^	3.4188 × 10^3^
	HBA Variant1	4.0870 × 10^3^	9.3854 × 10^4^	5.0798 × 10^3^	3.7310 × 10^3^
	HBA Variant2	3.9699 × 10^3^	4.9022 × 10^4^	4.4602 × 10^3^	3.5150 × 10^3^
	HBA Variant3	4.2053 × 10^3^	6.4014 × 10^4^	4.6087 × 10^3^	3.7526 × 10^3^
	HBA Variant4	3.6998 × 10^3^	1.8153 × 10^4^	4.1499 × 10^3^	3.4594 × 10^3^
	HBA Variant5	3.6399 × 10^3^	1.3522 × 10^4^	3.9809 × 10^3^	3.4785 × 10^3^
F29	HBA	5.4823 × 10^3^	1.7973 × 10^6^	1.0828 × 10^4^	4.0879 × 10^3^
	HBA Variant1	5.1122 × 10^3^	2.3084 × 10^5^	6.0240 × 10^3^	4.3307 × 10^3^
	HBA Variant2	5.1476 × 10^3^	4.0645 × 10^5^	7.4233 × 10^3^	4.1184 × 10^3^
	HBA Variant3	4.7388 × 10^3^	1.2051 × 10^5^	5.5547 × 10^3^	4.1877 × 10^3^
	HBA Variant4	4.7174 × 10^3^	2.0623 × 10^5^	5.8894 × 10^3^	3.8441 × 10^3^
	HBA Variant5	4.5941 × 10^3^	1.4672 × 10^5^	5.4513 × 10^3^	3.8514 × 10^3^
F30	HBA	3.9299 × 10^6^	5.6959 × 10^12^	1.2116 × 10^7^	1.4320 × 10^6^
	HBA Variant1	8.4490 × 10^6^	5.9644 × 10^13^	3.4072 × 10^7^	1.7421 × 10^6^
	HBA Variant2	6.6050 × 10^6^	1.4232 × 10^13^	1.6934 × 10^7^	2.1936 × 10^6^
	HBA Variant3	1.1878 × 10^7^	1.5381 × 10^13^	1.9686 × 10^7^	5.7392 × 10^6^
	HBA Variant4	3.5455 × 10^6^	1.5670 × 10^12^	6.1212 × 10^6^	1.1612 × 10^6^
	HBA Variant5	3.4745 × 10^6^	1.1059 × 10^12^	6.1616 × 10^6^	1.8977 × 10^6^

## Appendix B

**Table A3:** Comparative experimental results with other excellent algorithms.

Function	Algorithm		Mean		Std		Max		Min
F1	HBA		2.0695 × 10^9^		9.6826 × 10^18^		1.3714 × 10^10^		6.4449 × 10^7^
	DOA		1.0438 × 10^11^		2.5244 × 10^20^		1.2827 × 10^11^		6.6255 × 10^10^
	GWO		1.2594 × 10^10^		2.2319 × 10^19^		2.1692 × 10^10^		3.4475 × 10^9^
	DBO		5.9421 × 10^9^		9.8738 × 10^19^		5.7089 × 10^10^		9.0608 × 10^8^
	MCEHBA		**3.0151 × 10^8^**		1.1446 × 10^17^		**1.3671 × 10^9^**		**5.8205 × 10^7^**
	SO		6.4625 × 10^8^		1.0172 × 10^17^		1.3921 × 10^9^		1.3060 × 10^8^
	WOA		2.1963 × 10^10^		1.9271 × 10^19^		3.5304 × 10^10^		1.3665 × 10^10^
F3	HBA		1.5706 × 10^5^		1.3487 × 10^9^		2.7029 × 10^5^		1.0058 × 10^5^
	DOA		2.3091 × 10^5^		8.3189 × 10^9^		4.8942 × 10^5^		1.1008 × 10^5^
	GWO		1.7940 × 10^5^		1.1015 × 10^9^		2.5563 × 10^5^		1.2113 × 10^5^
	DBO		2.7257 × 10^5^		5.2314 × 10^9^		4.7108 × 10^5^		1.5910 × 10^5^
	MCEHBA		**5.5508 × 10^4^**		**1.5894 × 10^8^**		**7.6003 × 10^4^**		**3.1126 × 10^4^**
	SO		1.6541 × 10^5^		2.4918 × 10^8^		1.9985 × 10^5^		1.3531 × 10^5^
	WOA		3.0980 × 10^5^		1.0300 × 10^10^		5.3352 × 10^5^		1.7710 × 10^5^
F4	HBA		7.4283 × 10^2^		1.7178 × 10^4^		1.0367 × 10^3^		5.5817 × 10^2^
	DOA		3.4009 × 10^4^		4.4147 × 10^7^		4.8306 × 10^4^		2.4124 × 10^4^
	GWO		1.6875 × 10^3^		6.6577 × 10^5^		4.2350 × 10^3^		8.4732 × 10^2^
	DBO		1.2434 × 10^3^		1.1671 × 10^5^		1.9619 × 10^3^		8.0600 × 10^2^
	MCEHBA		**7.0617 × 10^2^**		**5.9397 × 10^3^**		**9.1396 × 10^2^**		6.2034 × 10^2^
	SO		8.4528 × 10^2^		1.6074 × 10^4^		1.1310 × 10^3^		6.5909 × 10^2^
	WOA		5.3971 × 10^3^		1.9613 × 10^6^		8.0861 × 10^3^		3.1167 × 10^3^
F5	HBA		7.6667 × 10^2^		1.5566 × 10^3^		8.3910 × 10^2^		6.7183 × 10^2^
	DOA		1.1536 × 10^3^		1.6404 × 10^3^		1.2607 × 10^3^		1.0801 × 10^3^
	GWO		7.6005 × 10^2^		2.1647 × 10^3^		9.3831 × 10^2^		7.0265 × 10^2^
	DBO		9.7916 × 10^2^		1.2220 × 10^4^		1.1769 × 10^3^		7.7662 × 10^2^
	MCEHBA		7.7058 × 10^2^		1.9386 × 10^3^		8.5794 × 10^2^		6.8246 × 10^2^
	SO		7.2024 × 10^2^		1.5009 × 10^3^		7.9200 × 10^2^		6.6156 × 10^2^
	WOA		1.1240 × 10^3^		6.7551 × 10^3^		1.3454 × 10^3^		1.0081 × 10^3^
F6	HBA		6.3727 × 10^2^		4.3875 × 10^1^		6.5432 × 10^2^		6.2087 × 10^2^
	DOA		6.9656 × 10^2^		5.6702 × 10^1^		7.1091 × 10^2^		6.8242 × 10^2^
	GWO		6.2472 × 10^2^		4.1318 × 10^1^		6.4022 × 10^2^		6.1545 × 10^2^
	DBO		6.6527 × 10^2^		1.5776 × 10^2^		6.9550 × 10^2^		6.4523 × 10^2^
	MCEHBA		6.3717 × 10^2^		1.5688 × 10^2^		6.6948 × 10^2^		6.1700 × 10^2^
	SO		6.3021 × 10^2^		3.5214 × 10^1^		6.4034 × 10^2^		6.1780 × 10^2^
	WOA		6.9722 × 10^2^		1.4061 × 10^2^		7.2598 × 10^2^		6.7444 × 10^2^
F7	HBA		1.2973 × 10^3^		1.2179 × 10^4^		1.6039 × 10^3^		1.1025 × 10^3^
	DOA		1.9435 × 10^3^		6.5751 × 10^3^		2.0931 × 10^3^		1.8171 × 10^3^
	GWO		1.1638 × 10^3^		6.2606 × 10^3^		1.4355 × 10^3^		1.0261 × 10^3^
	DBO		1.4291 × 10^3^		2.4198 × 10^4^		1.8517 × 10^3^		1.1113 × 10^3^
	MCEHBA		1.1760 × 10^3^		5.4324 × 10^3^		**1.3022 × 10^3^**		**1.0176 × 10^3^**
	SO		1.1988 × 10^3^		4.6494 × 10^3^		1.3824 × 10^3^		1.0852 × 10^3^
	WOA		1.9229 × 10^3^		8.4357 × 10^3^		2.1126 × 10^3^		1.7596 × 10^3^
F8	HBA		1.0785 × 10^3^		2.7494 × 10^3^		1.1773 × 10^3^		9.5582 × 10^2^
	DOA		1.4712 × 10^3^		2.1400 × 10^3^		1.6063 × 10^3^		1.3955 × 10^3^
	GWO		1.0782 × 10^3^		4.1308 × 10^3^		1.3394 × 10^3^		9.8528 × 10^2^
	DBO		1.2838 × 10^3^		1.0788 × 10^4^		1.4265 × 10^3^		1.0698 × 10^3^
	MCEHBA		1.0580 × 10^3^		2.2009 × 10^3^		1.1339 × 10^3^		9.6571 × 10^2^
	SO		1.0275 × 10^3^		1.3184 × 10^3^		1.1254 × 10^3^		9.5484 × 10^2^
	WOA		1.4047 × 10^3^		3.6591 × 10^3^		1.5188 × 10^3^		1.2902 × 10^3^
F9	HBA		1.3767 × 10^4^		3.8521 × 10^7^		3.0583 × 10^4^		4.8583 × 10^3^
	DOA		3.7493 × 10^4^		2.4412 × 10^7^		4.5304 × 10^4^		2.6496 × 10^4^
	GWO		1.1363 × 10^4^		2.0975 × 10^7^		1.9915 × 10^4^		5.2280 × 10^3^
	DBO		2.5564 × 10^4^		6.1261 × 10^7^		4.2696 × 10^4^		1.5372 × 10^4^
	MCEHBA		1.1230 × 10^4^		3.0131 × 10^7^		2.5183 × 10^4^		4.0967 × 10^3^
	SO		7.6076 × 10^3^		1.2313 × 10^7^		1.5197 × 10^4^		2.3628 × 10^3^
	WOA		4.0029 × 10^4^		1.5322 × 10^8^		8.5441 × 10^4^		2.2259 × 10^4^
F10	HBA		9.7063 × 10^3^		3.2627 × 10^6^		1.3139 × 10^4^		5.2862 × 10^3^
	DOA		1.5243 × 10^4^		6.4593 × 10^5^		1.6674 × 10^4^		1.3936 × 10^4^
	GWO		8.5431 × 10^3^		3.7485 × 10^6^		1.4410 × 10^4^		6.0348 × 10^3^
	DBO		1.2748 × 10^4^		4.5587 × 10^6^		1.5436 × 10^4^		8.8599 × 10^3^
	MCEHBA		**8.4038 × 10^3^**		1.3621 × 10^6^		**1.0923 × 10^4^**		6.2255 × 10^3^
	SO		9.5753 × 10^3^		8.3289 × 10^6^		1.5049 × 10^4^		5.7094 × 10^3^
	WOA		1.3379 × 10^4^		1.1311 × 10^6^		1.5296 × 10^4^		1.0445 × 10^4^
F11	HBA		1.9376 × 10^3^		4.5137 × 10^5^		4.3160 × 10^3^		1.4191 × 10^3^
	DOA		1.8455 × 10^4^		2.7414 × 10^7^		2.8226 × 10^4^		8.0883 × 10^3^
	GWO		7.5577 × 10^3^		8.6312 × 10^6^		1.3895 × 10^4^		3.0690 × 10^3^
	DBO		5.2983 × 10^3^		1.8423 × 10^7^		2.1878 × 10^4^		1.8497 × 10^3^
	MCEHBA		**1.4878 × 10^3^**		**1.1965 × 10^4^**		**1.8242 × 10^3^**		**1.3257 × 10^3^**
	SO		3.8917 × 10^3^		3.4065 × 10^6^		1.0371 × 10^4^		2.1241 × 10^3^
	WOA		1.0126 × 10^4^		1.0178 × 10^7^		1.7890 × 10^4^		4.9503 × 10^3^
F12	HBA		3.6192 × 10^7^		7.0996 × 10^14^		1.0180 × 10^8^		4.7769 × 10^6^
	DOA		5.8910 × 10^10^		4.2124 × 10^20^		1.0692 × 10^11^		2.3828 × 10^10^
	GWO		1.2380 × 10^9^		2.2000 × 10^18^		6.2847 × 10^9^		5.2333 × 10^7^
	DBO		8.1675 × 10^8^		3.3697 × 10^17^		2.9919 × 10^9^		1.5457 × 10^8^
	MCEHBA		**1.2762 × 10^7^**		**7.9364 × 10^13^**		**3.8917 × 10^7^**		**1.8567 × 10^6^**
	SO		7.9088 × 10^7^		6.6592 × 10^15^		4.3995 × 10^8^		1.5001 × 10^7^
	WOA		4.8373 × 10^9^		3.0510 × 10^18^		7.7991 × 10^9^		1.8251 × 10^9^
F13	HBA		1.0076 × 10^5^		3.4754 × 10^9^		2.1554 × 10^5^		2.2365 × 10^4^
	DOA		2.3085 × 10^10^		1.5727 × 10^20^		5.9541 × 10^10^		4.1498 × 10^9^
	GWO		3.6060 × 10^8^		8.4572 × 10^17^		5.1473 × 10^9^		4.5409 × 10^6^
	DBO		8.2913 × 10^7^		8.2025 × 10^15^		3.0945 × 10^8^		1.2640 × 10^5^
	MCEHBA		**1.2486 × 10^4^**		**6.2022 × 10^7^**		**3.9766 × 10^4^**		**3.9028 × 10^3^**
	SO		3.0123 × 10^5^		1.0623 × 10^11^		1.8065 × 10^6^		5.0275 × 10^4^
	WOA		5.5104 × 10^8^		1.2157 × 10^17^		1.7401 × 10^9^		7.6550 × 10^7^
F14	HBA		2.6441 × 10^5^		2.8387 × 10^10^		7.5839 × 10^5^		4.5580 × 10^4^
	DOA		1.4532 × 10^7^		5.4954 × 10^14^		8.5563 × 10^7^		3.3523 × 10^4^
	GWO		1.8488 × 10^6^		2.7895 × 10^12^		5.2927 × 10^6^		1.9769 × 10^5^
	DBO		3.9729 × 10^6^		1.8197 × 10^13^		1.7824 × 10^7^		5.9855 × 10^4^
	MCEHBA		**6.3779 × 10^4^**		**4.1637 × 10^9^**		**3.1160 × 10^5^**		**3.7074 × 10^3^**
	SO		5.1979 × 10^5^		2.6820 × 10^11^		2.7332 × 10^6^		7.5631 × 10^4^
	WOA		7.9599 × 10^6^		5.7445 × 10^13^		3.9736 × 10^7^		4.9366 × 10^5^
F15	HBA		4.0678 × 10^4^		2.2019 × 10^9^		2.4045 × 10^5^		5.0204 × 10^3^
	DOA		3.4611 × 10^9^		1.8443 × 10^19^		1.8913 × 10^10^		4.5036 × 10^7^
	GWO		1.9239 × 10^7^		6.3586 × 10^14^		7.9121 × 10^7^		5.6575 × 10^4^
	DBO		4.8553 × 10^6^		2.4732 × 10^14^		7.2855 × 10^7^		5.9004 × 10^4^
	MCEHBA		**9.1818 × 10^3^**		**3.9379 × 10^7^**		**2.0357 × 10^4^**		**2.0441 × 10^3^**
	SO		4.6860 × 10^4^		8.5759 × 10^8^		1.1708 × 10^5^		4.2541 × 10^3^
	WOA		7.4570 × 10^7^		8.6787 × 10^15^		4.5828 × 10^8^		7.6281 × 10^5^
F16	HBA		3.5708 × 10^3^		4.5209 × 10^5^		5.8189 × 10^3^		2.6745 × 10^3^
	DOA		7.0838 × 10^3^		1.3451 × 10^6^		9.7159 × 10^3^		4.8215 × 10^3^
	GWO		3.4694 × 10^3^		2.4019 × 10^5^		5.0294 × 10^3^		2.5597 × 10^3^
	DBO		4.9771 × 10^3^		5.2101 × 10^5^		6.6480 × 10^3^		3.7289 × 10^3^
	MCEHBA		**3.2015 × 10^3^**		1.9294 × 10^5^		**4.1826 × 10^3^**		**2.3215 × 10^3^**
	SO		3.4092 × 10^3^		1.6490 × 10^5^		4.3649 × 10^3^		2.6668 × 10^3^
	WOA		6.2405 × 10^3^		8.8968 × 10^5^		9.0786 × 10^3^		4.0811 × 10^3^
F17	HBA		3.2996 × 10^3^		1.2072 × 10^5^		4.1872 × 10^3^		2.7605 × 10^3^
	DOA		5.4714 × 10^3^		2.2282 × 10^6^		9.3809 × 10^3^		3.6994 × 10^3^
	GWO		3.1178 × 10^3^		1.8199 × 10^5^		4.5644 × 10^3^		2.5062 × 10^3^
	DBO		4.1529 × 10^3^		2.3130 × 10^5^		5.4883 × 10^3^		3.3170 × 10^3^
	MCEHBA		**3.0752 × 10^3^**		1.6050 × 10^5^		3.9915 × 10^3^		**2.3002 × 10^3^**
	SO		3.2381 × 10^3^		9.9343 × 10^4^		3.8158 × 10^3^		2.5248 × 10^3^
	WOA		4.7223 × 10^3^		4.8338 × 10^5^		7.0092 × 10^3^		3.7371 × 10^3^
F18	HBA		2.1217 × 10^6^		2.5551 × 10^12^		5.8486 × 10^6^		1.7950 × 10^5^
	DOA		3.5250 × 10^7^		1.7680 × 10^15^		1.8717 × 10^8^		1.8303 × 10^6^
	GWO		8.7644 × 10^6^		8.2128 × 10^13^		3.5027 × 10^7^		1.1957 × 10^6^
	DBO		1.0367 × 10^7^		1.1557 × 10^14^		4.7302 × 10^7^		5.9310 × 10^5^
	MCEHBA		**3.5468 × 10^5^**		**8.6724 × 10^10^**		**1.1641 × 10^6^**		**8.2333 × 10^4^**
	SO		4.8839 × 10^6^		1.4405 × 10^13^		1.9429 × 10^7^		7.4322 × 10^5^
	WOA		5.1177 × 10^7^		1.3608 × 10^15^		1.2894 × 10^8^		5.9922 × 10^6^
F19	HBA		2.8039 × 10^4^		2.7450 × 10^8^		7.9135 × 10^4^		2.8711 × 10^3^
	DOA		1.5286 × 10^9^		3.1373 × 10^18^		6.5365 × 10^9^		4.0989 × 10^7^
	GWO		9.3333 × 10^6^		3.8836 × 10^14^		9.9661 × 10^7^		3.0220 × 10^4^
	DBO		5.4440 × 10^6^		5.5496 × 10^13^		3.0609 × 10^7^		1.0943 × 10^4^
	MCEHBA		**2.2402 × 10^4^**		**8.1896 × 10^7^**		**3.9542 × 10^4^**		3.3165 × 10^3^
	SO		8.8793 × 10^4^		1.5720 × 10^10^		5.2019 × 10^5^		1.0263 × 10^4^
	WOA		1.8582 × 10^7^		2.0438 × 10^14^		5.4746 × 10^7^		2.3078 × 10^6^
F20	HBA		3.1633 × 10^3^		1.4159 × 10^5^		3.9113 × 10^3^		2.5381 × 10^3^
	DOA		4.0864 × 10^3^		1.9512 × 10^5^		4.7238 × 10^3^		3.0278 × 10^3^
	GWO		3.0793 × 10^3^		2.6499 × 10^5^		4.5893 × 10^3^		2.4050 × 10^3^
	DBO		3.8803 × 10^3^		1.4151 × 10^5^		4.4910 × 10^3^		2.9201 × 10^3^
	MCEHBA		**3.0766 × 10^3^**		**1.0183 × 10^5^**		**3.6793 × 10^3^**		2.4521 × 10^3^
	SO		3.0955 × 10^3^		1.2972 × 10^5^		3.8858 × 10^3^		2.4166 × 10^3^
	WOA		3.8920 × 10^3^		1.0398 × 10^5^		4.7996 × 10^3^		3.5111 × 10^3^
F21	HBA		2.5451 × 10^3^		3.0189 × 10^3^		2.6801 × 10^3^		2.4574 × 10^3^
	DOA		3.0837 × 10^3^		7.0114 × 10^3^		3.2242 × 10^3^		2.9423 × 10^3^
	GWO		2.5619 × 10^3^		4.9131 × 10^3^		2.7780 × 10^3^		2.4641 × 10^3^
	DBO		2.9001 × 10^3^		4.9919 × 10^3^		3.1072 × 10^3^		2.7719 × 10^3^
	MCEHBA		2.5319 × 10^3^		**2.3959 × 10^3^**		2.6503 × 10^3^		2.4635 × 10^3^
	SO		2.5134 × 10^3^		2.4706 × 10^3^		2.6238 × 10^3^		2.4386 × 10^3^
	WOA		3.0859 × 10^3^		1.3253 × 10^4^		3.3909 × 10^3^		2.8869 × 10^3^
F22	HBA		1.1000 × 10^4^		4.3324 × 10^6^		1.3129 × 10^4^		2.3968 × 10^3^
	DOA		1.6487 × 10^4^		1.2901 × 10^6^		1.8579 × 10^4^		1.3293 × 10^4^
	GWO		9.9687 × 10^3^		2.4335 × 10^6^		1.7011 × 10^4^		7.9810 × 10^3^
	DBO		1.3687 × 10^4^		5.1047 × 10^6^		1.7422 × 10^4^		1.0563 × 10^4^
	MCEHBA		**8.4953 × 10^3^**		1.4123 × 10^7^		**1.2723 × 10^4^**		2.4037 × 10^3^
	SO		1.1245 × 10^4^		5.7034 × 10^6^		1.6743 × 10^4^		7.3617 × 10^3^
	WOA		1.4553 × 10^4^		1.3447 × 10^6^		1.6379 × 10^4^		1.2222 × 10^4^
F23	HBA		3.0746 × 10^3^		5.3817 × 10^3^		3.2255 × 10^3^		2.9554 × 10^3^
	DOA		4.3155 × 10^3^		5.0739 × 10^4^		4.8585 × 10^3^		3.9820 × 10^3^
	GWO		3.0760 × 10^3^		1.1909 × 10^4^		3.3819 × 10^3^		2.9284 × 10^3^
	DBO		3.5518 × 10^3^		2.3814 × 10^4^		3.9166 × 10^3^		3.2630 × 10^3^
	MCEHBA		**3.0670 × 10^3^**		4.1148 × 10^3^		**3.2254 × 10^3^**		2.9697 × 10^3^
	SO		3.1065 × 10^3^		3.4693 × 10^3^		3.2582 × 10^3^		2.9658 × 10^3^
	WOA		3.8924 × 10^3^		4.1123 × 10^4^		4.4624 × 10^3^		3.5643 × 10^3^
F24	HBA		3.5167 × 10^3^		1.4935 × 10^5^		4.3594 × 10^3^		3.0860 × 10^3^
	DOA		4.4084 × 10^3^		6.8246 × 10^4^		5.0670 × 10^3^		3.9802 × 10^3^
	GWO		3.2099 × 10^3^		7.9998 × 10^3^		3.4637 × 10^3^		3.1145 × 10^3^
	DBO		3.6900 × 10^3^		2.1060 × 10^4^		3.9248 × 10^3^		3.2991 × 10^3^
	MCEHBA		6.7454 × 10^3^		**8.5570 × 10^−25^**		6.7454 × 10^3^		6.7454 × 10^3^
	SO		3.2313 × 10^3^		5.1095 × 10^3^		3.3725 × 10^3^		3.1187 × 10^3^
	WOA		3.9003 × 10^3^		2.3072 × 10^4^		4.1971 × 10^3^		3.6089 × 10^3^
F25	HBA		3.2809 × 10^3^		1.6262 × 10^4^		3.6830 × 10^3^		3.1145 × 10^3^
	DOA		1.4334 × 10^4^		3.6255 × 10^6^		1.8531 × 10^4^		1.0860 × 10^4^
	GWO		3.8879 × 10^3^		2.1725 × 10^5^		5.5553 × 10^3^		3.4002 × 10^3^
	DBO		3.5123 × 10^3^		1.2209 × 10^5^		4.8302 × 10^3^		3.1551 × 10^3^
	MCEHBA		**3.2339 × 10^3^**		**4.1405 × 10^3^**		**3.3574 × 10^3^**		**3.1021 × 10^3^**
	SO		3.3542 × 10^3^		1.3627 × 10^4^		3.6467 × 10^3^		3.1538 × 10^3^
	WOA		5.0654 × 10^3^		3.6823 × 10^5^		6.6509 × 10^3^		4.0059 × 10^3^
F26	HBA		7.8675 × 10^3^		2.0682 × 10^6^		1.1250 × 10^4^		6.2028 × 10^3^
	DOA		1.6630 × 10^4^		8.9848 × 10^5^		1.8678 × 10^4^		1.4722 × 10^4^
	GWO		7.1346 × 10^3^		4.9210 × 10^5^		9.2298 × 10^3^		6.2458 × 10^3^
	DBO		1.0873 × 10^4^		1.2957 × 10^6^		1.3326 × 10^4^		8.6845 × 10^3^
	MCEHBA		7.7973 × 10^3^		5.8060 × 10^6^		1.1323 × 10^4^		**3.9799 × 10^3^**
	SO		7.9832 × 10^3^		4.3799 × 10^5^		9.5427 × 10^3^		6.7647 × 10^3^
	WOA		1.4719 × 10^4^		1.5514 × 10^6^		1.7939 × 10^4^		1.2144 × 10^4^
F27	HBA		4.2178 × 10^3^		3.1739 × 10^5^		5.4107 × 10^3^		3.5331 × 10^3^
	DOA		5.2141 × 10^3^		5.1956 × 10^5^		7.4950 × 10^3^		4.2194 × 10^3^
	GWO		3.7172 × 10^3^		8.9873 × 10^3^		3.9398 × 10^3^		3.5325 × 10^3^
	DBO		4.0118 × 10^3^		6.4224 × 10^4^		4.4752 × 10^3^		3.4333 × 10^3^
	MCEHBA		3.8229 × 10^3^		3.8791 × 10^4^		4.2638 × 10^3^		**3.4168 × 10^3^**
	SO		3.8177 × 10^3^		1.1290 × 10^4^		3.9954 × 10^3^		3.5502 × 10^3^
	WOA		4.9443 × 10^3^		3.8168 × 10^5^		6.3568 × 10^3^		3.9880 × 10^3^
F28	HBA		3.7229 × 10^3^		5.1602 × 10^4^		4.3789 × 10^3^		3.4129 × 10^3^
	DOA		1.1909 × 10^4^		3.0423 × 10^6^		1.6718 × 10^4^		9.1833 × 10^3^
	GWO		4.6919 × 10^3^		2.2993 × 10^5^		5.8259 × 10^3^		3.8690 × 10^3^
	DBO		6.5732 × 10^3^		4.6599 × 10^6^		1.0470 × 10^4^		3.7894 × 10^3^
	MCEHBA		**3.6634 × 10^3^**		**1.5300 × 10^4^**		**3.9826 × 10^3^**		**3.3781 × 10^3^**
	SO		4.4034 × 10^3^		2.3813 × 10^5^		5.5741 × 10^3^		3.6568 × 10^3^
	WOA		6.2543 × 10^3^		2.4056 × 10^5^		7.2452 × 10^3^		5.1703 × 10^3^
F29	HBA		5.2623 × 10^3^		9.7129 × 10^5^		8.6210 × 10^3^		4.0839 × 10^3^
	DOA		1.8576 × 10^4^		2.1655 × 10^8^		8.7528 × 10^4^		7.7126 × 10^3^
	GWO		5.0178 × 10^3^		1.5663 × 10^5^		6.2638 × 10^3^		4.4799 × 10^3^
	DBO		6.4521 × 10^3^		1.1656 × 10^6^		9.8359 × 10^3^		4.7972 × 10^3^
	MCEHBA		**4.6496 × 10^3^**		1.8310 × 10^5^		**5.5785 × 10^3^**		**3.8298 × 10^3^**
	SO		5.1542 × 10^3^		1.1580 × 10^5^		5.9198 × 10^3^		4.3836 × 10^3^
	WOA		9.1519 × 10^3^		2.6801 × 10^6^		1.2052 × 10^4^		6.5662 × 10^3^
F30	HBA		4.8784 × 10^6^		1.5349 × 10^13^		1.9193 × 10^7^		1.4177 × 10^6^
	DOA		2.4332 × 10^9^		4.4774 × 10^18^		1.0983 × 10^10^		3.1077 × 10^8^
	GWO		1.9402 × 10^8^		1.1396 × 10^16^		6.0845 × 10^8^		6.9103 × 10^7^
	DBO		5.8819 × 10^7^		4.2998 × 10^15^		3.4509 × 10^8^		9.7465 × 10^6^
	MCEHBA		**3.7144 × 10^6^**		**2.2500 × 10^12^**		**9.1977 × 10^6^**		1.7939 × 10^6^
	SO		1.6104 × 10^7^		8.3876 × 10^13^		3.8096 × 10^7^		5.1475 × 10^6^
	WOA		3.1454 × 10^8^		1.4429 × 10^16^		6.7171 × 10^8^		9.2151 × 10^7^
result		20/29		15/29		20/29		16/29	
